# Dynamics of technology emergence in innovation networks

**DOI:** 10.1038/s41598-023-50280-4

**Published:** 2024-01-16

**Authors:** Martin Ho, Henry C. W. Price, Tim S. Evans, Eoin O’Sullivan

**Affiliations:** 1 Centre for Science Technology and Innovation Policy, University of Cambridge, Cambridge, CB3 0HU UK; 2 Department of Engineering, University of Cambridge, Cambridge, CB3 0HU UK; 3 Centre for Complexity Science, Imperial College London, London, SW7 2AZ UK; 4 Theoretical Physics Group, Department of Physics, Imperial College London, London, SW7 2AZ UK

**Keywords:** Business strategy in drug development, Drug discovery, Drug development, Preclinical research, Translational research, Complex networks, Physics, Statistical physics, thermodynamics and nonlinear dynamics, Scientific data, Statistics

## Abstract

To create the next innovative product, participants in science need to understand which existing technologies can be combined, what new science must be discovered, and what new technologies must be invented. Knowledge of these often arrives by means of expert consensus or popularity metrics, masking key information on how intellectual efforts accumulate into technological progress. To address this shortcoming, we first present a method to establish a mathematical link between technological evolution and complex networks: a path of events that narrates innovation bottlenecks. Next, we quantify the position and proximity of documents to these innovation paths. The result is an innovation network that more exhaustively captures deterministic knowledge flows with respect to a marketed innovative product. Our dataset, containing over three million biomedical citations, demonstrates the possibility of quantifying the accumulation, speed, and division of labour in innovation over a sixty-year time horizon. The significance of this study includes the (i) use of a purpose-generated dataset showing causal paths from research to development to product; (ii) analysis of the innovation process as a directed acyclic graph; (iii) comparison between calendar time and network time; (iv) ordering of science funders along technology lifecycles; (v) quantification of innovative activities’ importance to an innovative outcome; and (vi) integration of publication, patent, clinical trial, regulatory data to study innovation holistically.

## Why do we need to understand the order of innovations?

We all know time flows linearly in one direction. On the other hand, innovation is historically one-directional but nonlinear^1^. Therefore, studies that present innovation events on a linear calendar timescale alone cannot represent the causality, importance, and convergence of innovation intermediaries. In multi-step reactions in chemistry, reactants do not jump straight to products; there are intermediaries with different activation energies and always a rate-determining step that the overall reaction cannot proceed faster than. Chemists often catalyse the rate-determining step to speed up the overall reaction. Likewise, an innovation process contains intermediary outputs, and we further propose that there are bottlenecks whose catalysis would accelerate the overall innovation process. Accordingly, we investigate:

In what *order* did individual technological breakthroughs occur to realise innovation outcomes? And, by extension, in what *order* did innovating entities support the most rate-limiting innovations along an order? To answer these questions, we prototype using a multilayer directed acyclic graph (DAG) to order scientific and technological precursors of innovation breakthroughs.

We propose methods to understand innovation order because contemporary analytical frameworks: phases and systems of innovation^1,2^, and contemporary methods: regression^3–7^ and conventional citation analyses^8–14^, may fall short of systematic causal explanations of complex, multi-phase innovations^15^.

Existing innovation frameworks often characterise innovation as phases, where innovations at the basic research phase flow into applied research, development, and application phases, either in one direction^16,17^ or multiple directions^1,2^. However, these flows were never measured at scale. In this study, we provide data proxy and network structure to account for the dynamics of innovation with reference to these frameworks.

In statistics, a correlation is observed when the regression results in a good data fit onto some line or curve. In econometrics and clinical statistics, causation is further established if other variables affecting the correlation are eliminated or corrected for. The latter involves estimating the difference in trends between (i) what would have happened if there is an intervention (e.g. policy or medical intervention) and (ii) what would have happened if there is no intervention (i.e. the counterfactual). In macroeconomic systems, because only either (i) or (ii) can be the reality at each time point, causal effects are estimated via the potential outcomes reasoning, which involves “as-if” randomly assigning samples into (i) or (ii), so that the *average* treatment effect is the difference between the *average* outcome in the treatment group and the *average* outcome in the control group. This reasoning attempts to simulate the random assignment of economic entities as if patients in a clinical trial are randomly assigned into treatment and control groups in a hospital. This attempt is known as a “natural experiment”. Innovation studies that apply regression typically estimate the causal relation between certain R &D funding regimes and certain socioeconomic outcomes, which involve technological emergence, with substantial *ceteris paribus*^3–7^.

Another strand of regression studies for innovation performs conditional probability statistics on citation databases^8–12^, offering a more direct route to interrogate innovation policy outcomes without artificial sample assignments. For instance, it is found that the US National Institutes of Health funded 29% of papers associated with 210 FDA new molecular entity approvals in 2010–2016^12^. However, regression alone cannot directly trace the emergence of technologies from first principles.

Meanwhile, the study of innovation through citation networks is directly based on the factual links between innovation records. The best-known approach is “main path analysis” proposed by Hummon and Doreian^13^ and, with variations, implemented in some popular analysis packages^14^ (see appendix F.3 for details). The weights used in main path analysis are formed by looking at all paths from a manually defined set of initial nodes (such as the first publications in the data set, sometimes all publications) to a manually defined set of final destination nodes, each path equally weighted. The method may fail because it relies on a single path rather than the inability to look at good paths for innovation^18^. Rather than deriving meaning from network geometry, many studies that rely on out-of-the-box main path analysis packages want to reduce the number of nodes in a citation network into a single chain of events to enable qualitative interpretation. Another characteristic relates to the reliance on manually defined keywords or datasets to demarcate the sample^19^. It is, therefore, unsurprising that main path analysis fails to identify technological trajectories, such as that of semiconductors^20^.

In summary, the two common approaches to the study of innovation have limitations. The regression approach is unsuitable for studying complex, long-term, multivariate, nonlinear systems such as technological emergence. This is because, in a complex system, regressions must confront trade-offs between adding more variables (in this paper’s case, millions of nodes) and losing degrees of freedom. Technically, a standard regression equation can include all the variables, but it is typically limited to one outcome variable and may struggle to trace or even define the various intermediate variables that are dependent on each other. Existing citation analyses describe technological emergence more closely but are restricted in terms of data - the manual definition of keywords is usually limited to publication and patent data, and there is an inability to find meaningful innovation paths robustly.

As a DAG contains a causally ordered chain of events, provided the network data is sufficient and relevant, from there, we can directly observe the causal path of input A to outcome B along with all causal intermediaries. Network science describes and analyses complex systems through abstraction, with nodes representing events/entities and edges representing a connection between a node pair. The network approach has been successful in deducing properties of real networks, such as the fat-tailed degree distribution (power law) and community behaviours (e.g. centrality) of entities^21,22^. Despite this success, there have been few attempts to deduce causal relations in multilayer networks, an aspect fundamental for understanding complex systems such as innovation^23^.

This paper shows how to represent innovation order and demonstrates how this can better our understanding of innovation from an evolutionary perspective. In “Data and methods” section outlines an original method of applying graph theory to derive information from multiple types of innovation data. In “Results” section presents and interprets results; “Discussion” section discusses economic insights from the use of longest path to order innovation data.

## Data and methods

Clinical approval data was obtained from the US Food and Drug Administration (FDA), the European Medicines Agency (EMA), and the UK Medicines and Healthcare products Regulatory Agency (MHRA). Clinical trial, patent, journal publication, and citations between these documents are from ClinicalTrials.gov, Lens.org^24^, Dimensions.ai^25^ respectively.

We capture the flow of innovation encoded in these documents through a network representation. Each node represents a single document, which is one of four types: an innovation outcome represented by regulatory authorisation, a clinical trial, a patent, or an academic publication. So, our networks are examples of what are called multilayer networks, for example, see^26^, as each type of node can be visualised as placed on a different layer, see fig. B.1 in appendix. The citations from one node *u* to another node *v* give us directed edges, written as (*u*, *v*), so our networks are examples of citation networks.

The networks we use all start from a single seed node, known as the source node, representing the regulatory marketing authorisation for one vaccine. We then grow the network using snowball sampling. That is, we follow the citations from the set of documents $\mathcal {V}(\ell )$ found at the $\ell$-th step of sampling, each citation giving us a new edge in our network, and whenever we find a new document this is added to the set of documents $\mathcal {V}(\ell +1)$ to be used in the next iteration of the sampling. Typically we stop after three steps. We then finish by adding edges from any node *v* in this last set of documents $\mathcal {V}(\ell =3)$ provided *v* cites a document *r* which is already in our network. We also augment this process at the first step in a number of ways, see section B.3 in appendix for details.

Usually, edges point backwards in time as each document can only cite an older document. However, for various reasons (discussed in section B.3 in appendix) there are some cases where this is not true in our data. This leads to the existence of a few cycles in our data. The final step in creating our network is to remove a small number of edges to ensure there are no cycles.

At the end of our process, our data is represented by a DAG, a directed acyclic graph, where the nodes always have two additional labels. First, we record which of the four types of documents a node represents. The second node label gives a single date, which we call the publication date: the priority date for patents, the start date for clinical trials, and the official publication date for an academic article.

A DAG has one special property that we will exploit. A path in a network is a sequence of distinct nodes, $\{u_0,u_1,\ldots ,u_\ell \}$, where each consecutive pair of nodes forms an edge so $(u_i,u_{i+1})$ is an edge representing the citation from document $u_i$ to document $u_{i+1}$. The length of a path, $\ell$, is the number of edges in the path (one less than the number of nodes). In a DAG, if two nodes are connected by at least one path, then we find that the longest paths (there can be more than one) are typically of a reasonable length and that they play an important role in the analysis of DAGs, as discussed in more detail in section F.2 in appendix. In particular, we will define the distance between pairs of nodes in a DAG to be equal to the length of the longest path between two nodes. This is quite different from analysis of other types of network where the existence of cycles means the longest path has little value, and it is the shortest path that plays an important role e.g. as in^26^.

The role of the longest paths in a DAG can be seen in two standard properties of their nodes. The height *h*(*v*) of a node *v* is the maximum distance from the source node (the seed authorisation document) to the node *v* while the depth *d*(*v*) is the maximum distance from node *v* to any of the sink nodes.

We argue that critical developments will lie on, or close to, a longest path. This is because longest paths in DAGs embedded in Lorentzian space-time are the closest approximation to the geodesics (see Discussion section for an analogy). Therefore, it is extremely useful to be able to look at documents that do not lie on one of the longest paths to the source node but, instead, lie on a path from source to sink that is one or two steps shorter than the longest path in the DAG. That is, we will also consider documents that are *close* to a longest path. To quantify what we mean by ‘close’ in this context, we define criticality *c*(*v*) for a node *v* as:

1 $$\begin{aligned} c(v)= h_\text{max}- h(v) - d(v). \end{aligned}$$

The criticality *c*(*v*) of a node *v* takes integer values between zero and the height of the DAG $h_\text{max}$ (this is the largest value of height or depth of any node). If *c*(*v*) is zero, node *v* lies on a longest path from the source node to a sink node. A node with criticality *c* lies on a path which is *c* steps shorter than the longest path. Thus, the criticality value of a node can be thought of as the distance of a node to one of the critical paths down which the key innovations flow.

**Figure 1:**
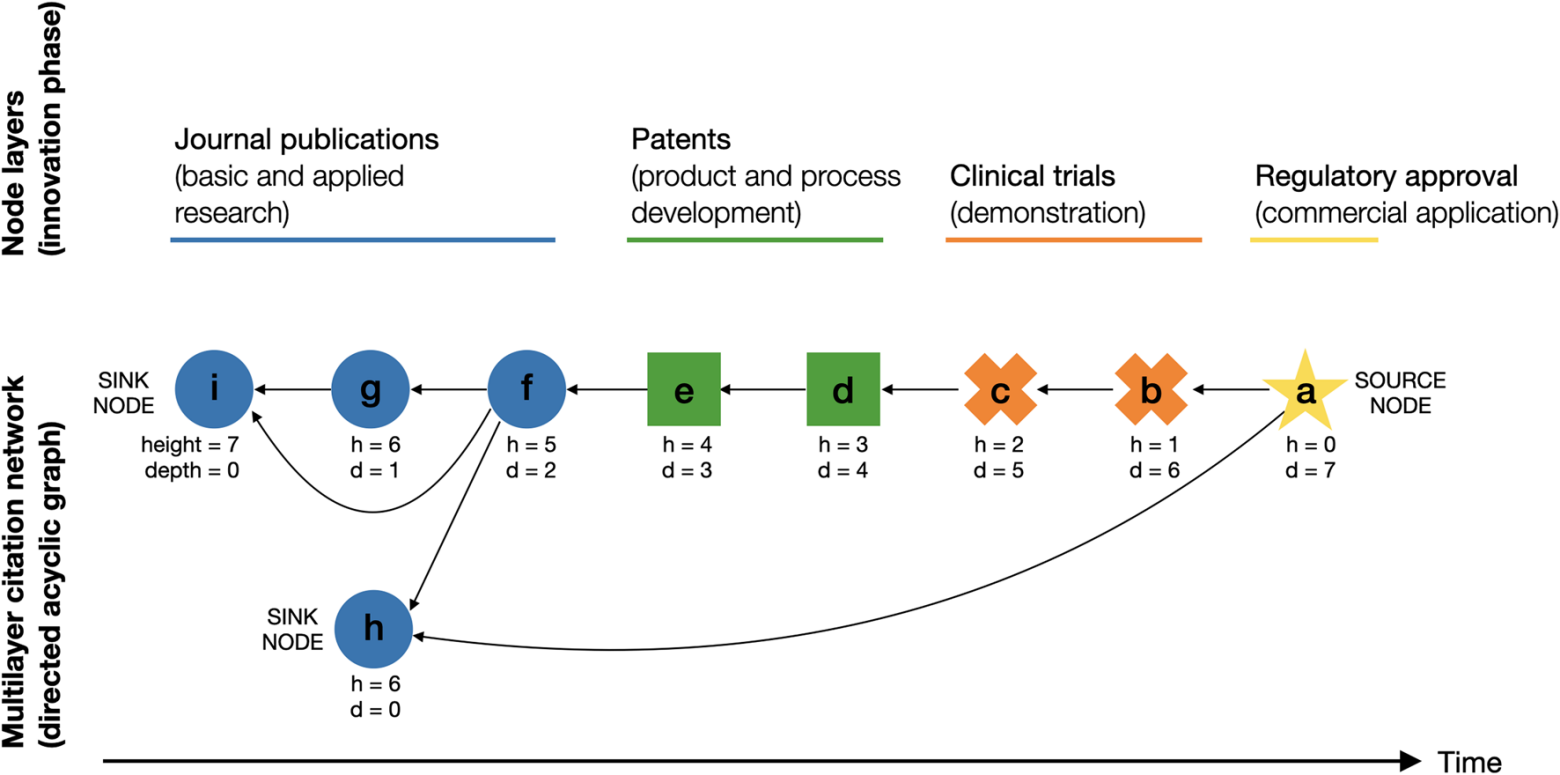
Conceptual framework for multilayer innovation network. Arrows represent direct citations from newer to older documents, embedding causality and the flow of time. Colours represent different data sources and approximate innovation phases. Only one source node with height 0 exists, but multiple sink nodes with depth 0 may exist. In this illustrative figure, the critical path is formed by nodes *abcdefgi* and represents the maximum distance between any two nodes in the graph. We further propose that the critical path in a multilayer innovation network represents a series of cumulative knowledge used for an innovation outcome. In contrast, any edge in a graph can be a shortest path, which might miss information on knowledge inheritance.

The novelty of our method is that we use the criticality values *c*(*v*) of nodes to find both the nodes on the longest paths but also important nodes lying on *near-longest paths* (small values of criticality). It is easy, therefore, for us to find other critical innovations which may have been missed by any method based on a single path, c.f. conventional main path analysis^14,18^ which always returns a single path (see section F.3 in appendix for details). The analytical methods are elaborated in section B of the appendix and illustrated in Fig. 1.

## Results

We use an original method (see “Data and methods” section) to move from six types of raw innovation data to produce citation networks which encode the multiplicity of innovation phases. We then perform statistical analysis on these networks to interrogate the accumulation, speed, and division labour in innovation.

### Descriptive statistics

Data on biomedical innovations is an excellent source, not only because the concept of translation is most established in medical research but also because new therapeutics are required by law to be reported and registered. In particular, we focus on eight vaccine approvals where there is excellent recent data available. The eight citation networks we study range in size from 15,282 nodes and 81,716 edges up to 153,446 nodes and 953,002 edges, see table B.2 in appendix.

### Critical innovation path narrates causality in innovation

**Figure 2:**
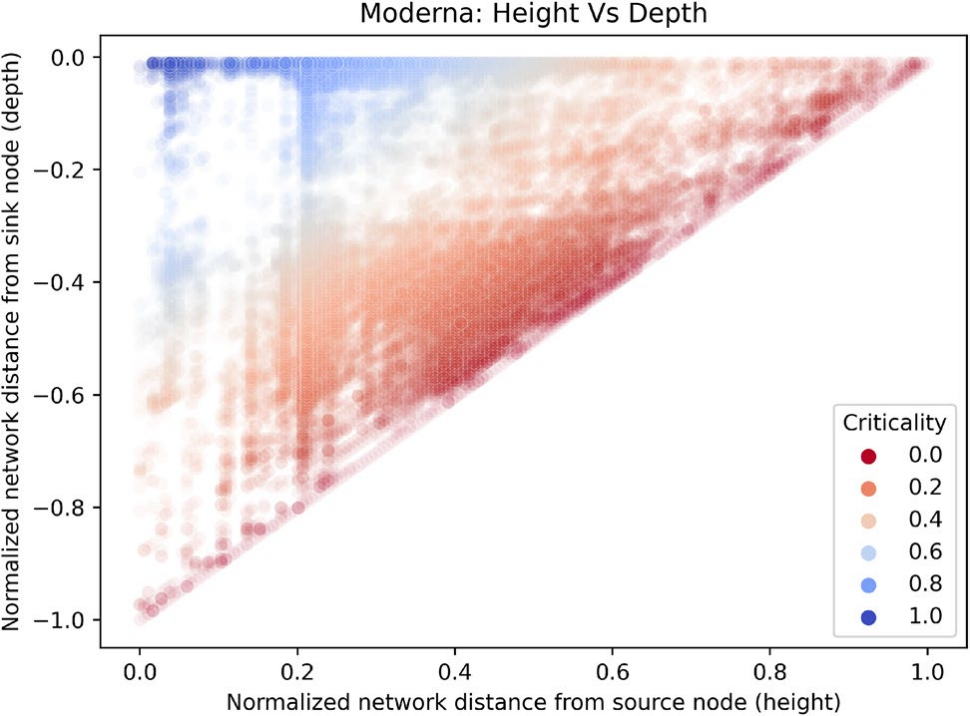
Critical innovation path represented by low criticality nodes. Illustrative data from Moderna COVID mRNA vaccine DAG showing the depth of nodes plotted as a function of height, both normalised by the height of the DAG $h_\text{max}$. The colour represents different values of criticality, again normalised by $h_\text{max}$, with 0.0 being the most critical and 1.0 the least critical. The diagonal of the plot are documents lying on one of the longest paths where $c = 0$. The complete set of nodes with assigned criticalities is available at 10.6084/m9.figshare.22155242.

Graphically, we follow Eq. (1) and plot depth as a function of height. We plot the data from the mRNA vaccine graph in Fig. 2 to illustrate: the bottom left node represents regulatory authorization, and the top right nodes represent the earliest nodes in the network. The diagonal represents nodes which lie on at least one longest path of the DAG, our critical innovation paths, while numerous sub-critical nodes populate the region above the diagonal. From the hue of the diagram, we also observe a cluster of non-critical innovations at the region with low height and low depth. We observe the same pattern in all eight DAGs, as shown in section D of the appendix. We set forth to inspect: (i) nodes that are critical, (ii) the order of critical nodes from oldest to newest, and (iii) nodes that are of lowest criticalities to test the theoretical equivalence between critical schedule path and longest network path.

**Figure 3:**
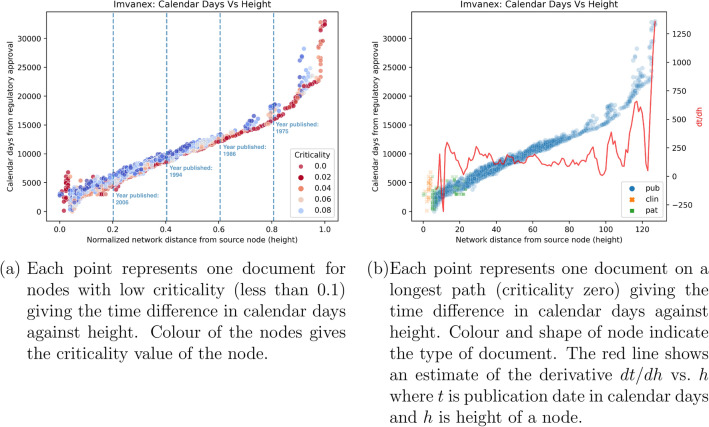
Time as a function of height for the Imvanex network. Height is normalised by the largest value so 0.0 is the regulatory approval of the Imvanex vaccine^46^ while 1.0 is for nodes at the largest network distance from the regulatory approval node. The time difference between the document publication date and the regulatory approval date is given in calendar days.

Looking at nodes whose criticality is strictly zero (i.e. most critical), in each DAG in Fig. 3, we see a mix of nodes representing publication, clinical trials, and regulatory authorisation. If we relax the criticality threshold to consider nodes whose criticality is below 19.5% of the maximum height, we begin to see many more publications, a few more clinical trials, and a few patents in this relaxed critical path region. The order of the critical path, moving from high to low height nodes, always proceeds from publications, intertwined with a much smaller number of patents if in the version with the 19.5% threshold, followed by phase 1, 2, and 3 clinical trials, before ending with the regulatory authorisation. This sequence generally proceeds from basic and applied research (publications), product and process development (patents), demonstration (clinical trials), and commercial application (regulatory authorisations).

A closer look at the critical path nodes unveils a logical sequence of technical progression. For instance, the Moderna mRNA vaccine DAG has its longest paths formed by early attempts to apply mRNA as an influenza vaccine platform^27,28^, using liposomal delivery system to enhance the expression kinetics of mRNA vaccine^29–31^, methylation to enhance *in vivo* antigen expression^32^, the phases 1-3 clinical trials of mRNA COVID vaccines (NCT04283461, NCT04796896, NCT04847050, NCT04470427), and finally the FDA emergency use authorisation letters^33^ events that the scientific literature is well aware of^34,35^. In addition, the longest path of the same DAG also identified critical discoveries that may have been overlooked: mRNA post-transcriptional modification mechanisms^36–41^ and early basic research about the potential to modify RNA to evade detection by toll-like receptors^42–44^.

We are also interested in the identity of non-critical nodes. Having low criticality in a DAG does not mean the innovation is unimportant; it means events are not rate-limiting and can be perhaps parallelised. Empirically, in the BioNTech/Pfizer COVID vaccine DAG, for example, nearly all reviewed nodes with low criticality are either clinical research about prevalence and risk factors for diseases non-specific to COVID. Low criticality events are likely non-critical to the approval of the vaccine by regulatory agency and, in this example, used to facilitate the design of clinical protocols.

Since we can measure the criticality of every innovation event within our network, we can also compute the propensity of different research agencies to fund critical innovations (table E.1 in appendix).

### Calendar time against height reveals innovation speed

The order inherent in a DAG gives a natural “clock” for the innovation process captured by our citation network. It is interesting to see how this network order compares against calendar time. To see this, we plotted the number of days between a document’s date and the final regulatory authorisation against the height of that document in Fig. 3. This shows that calendar date is strongly correlated with network order, but the relationship is non-linear. Broadly speaking, the smallest calendar day at every height are nodes on the longest path (i.e. they are nodes with 0 criticality), but why is the rate of change, shown by the red line in Fig. 3, non-constant?

Time and network order in a citation network proceed in the same direction. This is because new documents can only cite older documents and, similarly, innovation is cumulative^45^. However, their unit of progression differ: time proceeds in evenly spaced seconds or days, whereas network order proceeds in citation steps that are non-equidistant. As an analogy, an innovation “clock” is one containing ticks that are spaced out differently. The latter means that the time gap and frequency of citations can both increase and decrease over the course of an innovation lifecycle.

Visually, Fig. 3a suggests the publication date is rising at a constant rate for most critical nodes, but the rate increases for documents with a normalised height close to 1.0. We have tried to estimate the rate of change of publication date against height in Fig. 3b by smoothing the data for those nodes on a longest path. On small scales, the change in height with calendar time fluctuates as seen in the red line in Fig. 3b. On a larger scale, the trend overall shows that height and time are reasonably correlated. This could show that network order provides an alternative measure of innovation progress compared to calendar time (section F.7 in appendix).

The first finding is that the order of node types along the critical path in Fig. 3b shows a clear progression of publications (basic and applied research) to patents (product and process development) to clinical trials (demonstration). However, if we also consider non-zero criticality nodes, we start to see more overlaps between node types. Phenomenologically, this shows the “stochastic” search for innovation often involves feedbacks across different document types – heeding to Kline and Rosenberg’s “chain-linked” model of innovation^1^. On the other hand, when it comes to critical innovation bottlenecks, a clear progression from research to development to demonstration is observed, agreeing with the “linear” model of innovation^16,17^.

The second finding is the negative rates of change in Fig. 3b. The existence of forward (future) and backward (past) citations is due to the patenting process often spanning several years. We found that due to interactions between patent applicants and examiners during patent prosecution, the patent document may be updated with new references. We use the initial patent submission date as our patent publication date. A year or two into the patent process, a recent paper can be added to the application, one that was published after the patent was submitted. As a result, a patent may cite forward in time as well as the logically acceptable backwards in time. We could use the patent award date as our patent publication date, which would solve the problem with the example just given. However, we now run into problems with documents that cite a patent that is not yet approved, which is a critical part of the innovation process. This again illustrates why our using the height of a node in our citation network can be a more consistent record of the logical order in the innovation process.

The third and most interesting finding is that innovation accelerates as a technology matures.If each edge is assumed to be the least publishable unit of knowledge, the decreasing time taken to move up one unit of height means the “innovation clock” is speeding up. The rates of change for critical patents and clinical trials fluctuate between 300 and -300 days, with the negative values indicating the problems of using a single publication date for patents, as these are revised over the several years it takes for a patent to be approved. On the other hand, it takes 50-1300 days for height to increase by one in the early critical journal publications, whereas more recent critical publications, those closer to the regulatory approval, have one year for a height increase of one, indicating an increasing rate of innovation towards the later stage. The observed fluctuations at the beginning and end of the networks might stem from data limitations. These limitations include significant shifts in citation behaviours (changes in node type), subject domains becoming well-defined and focused, and sparser data in the past. The snowball sampling (appendix B.3) method used for network generation also causes data truncation as we have set a predefined step limit on the network’s size.

**Figure 4:**
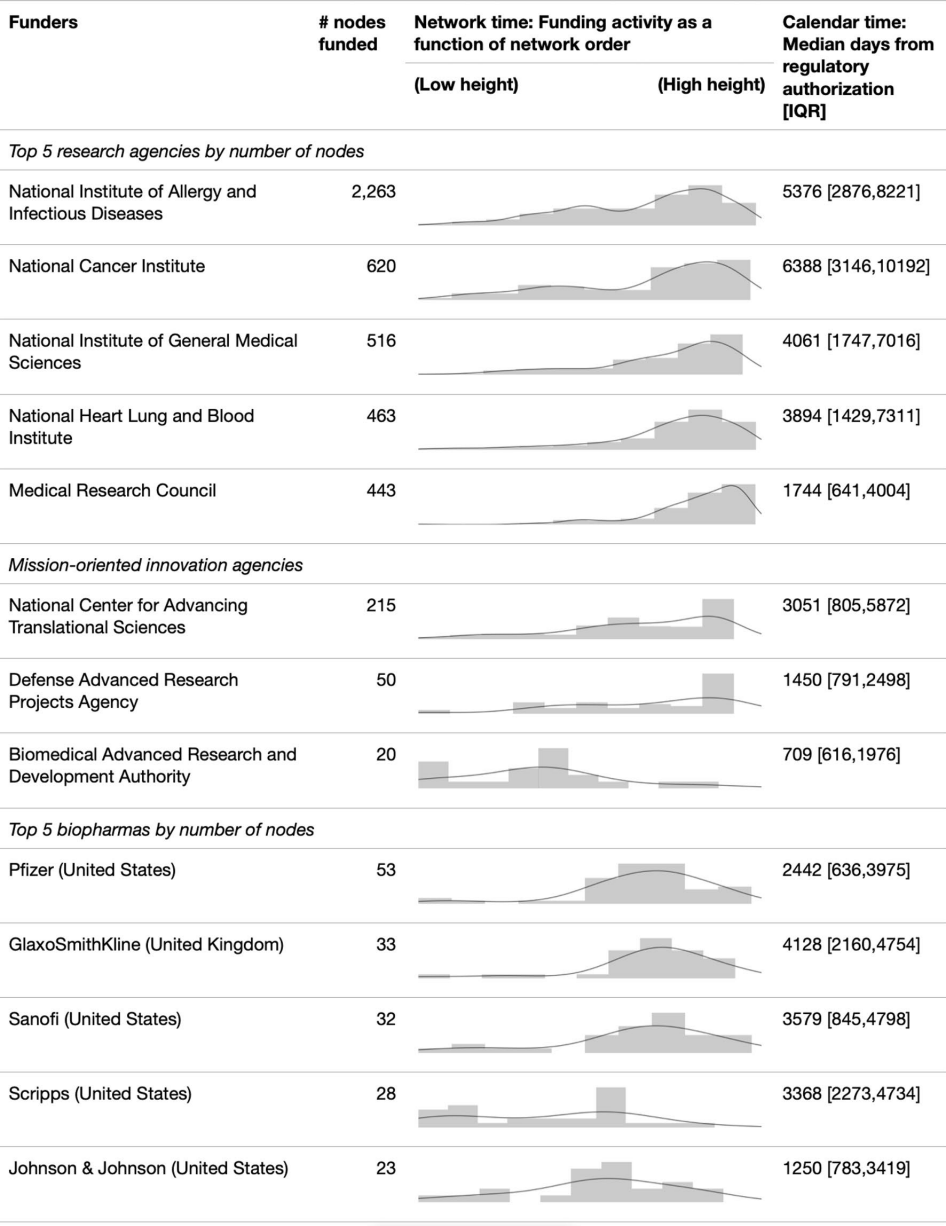
Funding activities as a function of network height. Illustrative data from Novavax vaccine. Kernel density estimations of heights of nodes associated with the funders. All entries are normalised on the same scale. Low height means a document has low citation distance from the regulatory approval. Low height typically, but not always, means it is at the late stage of innovation; the converse is true. Days are the calendar days between the regulatory approval and documents funded by the funders. Some funders were involved in the manufacturing and procurement of vaccines, but these data are not available in the network; it is therefore likely that their actual funding activity curves are more skewed to the left.

An innovation dynamic we observe is that the first derivative of calendar days with respect to network height increases with network height. The conceptual framework of Dosi^47^ and case studies of Auerswald and Branscomb^48^ provide a plausible explanation for this phenomenon: At the nascent stage of technology, the organisation of innovation (nodes and edges) was largely random. Public research provides the necessary technology “push” to de-risk innovations required for a solid business case by reducing asymmetries of scientific information and motivation. As a technology matures, the accumulation of technological knowledge and refinement of the direction of search – through clarifications of research purpose and consumer demands, and increased R&D funding – likely facilitate more frequent and targeted innovations towards the vaccine. This is because the innovation actors involved in the vaccine have become more adept at identifying and exploiting emerging technology’s opportunities. This handover from public to private sector is confirmed by Fig. 4 and discussed in the next section. Across the eight vaccines (Fig. D.3 in the Appendix), making critical progress at later innovation phases always takes less time.

### Division of innovation labour is quantifiable via network height

Using the findings above, we demonstrate another real-world utility of innovation order. We portray the frequency of innovator funding as a function of network height to discern the innovation phases entities are supporting (section B.5 in appendix for methods). Figure 4 shows the top five funders by number of nodes funded, three mission-oriented innovation agencies (entities that specifically fund frontier innovations to attain specific goals^49^), and the top five pharmaceuticals by number of nodes funded. We observe that the largest funders tend to occupy lower height, or early-stage; pharmaceuticals fund a mix of mid- and late-stage documents; whereas mission-oriented innovation agencies are generally more evenly spread across different innovation stages. Looking at calendar time, the median days of mission-oriented agencies and pharmaceuticals (2-19 years) are much closer to regulatory approval than large funders are (10-27 years). This may indicate the strategies and division of labour among innovation entities: Larger funders fund basic and risk-averse research, mission-oriented agencies initiate high-risk research and translate discoveries to other funders, and pharmaceuticals playing their obvious commercialization role at the later stages. Although we do not know whether this division of labour is deliberate or a result of their funding agenda, compared to innovation input data such as R &D spending by agency^50^, our results reflect inter-agency coordination in innovation.

### Validation

**Figure 5:**
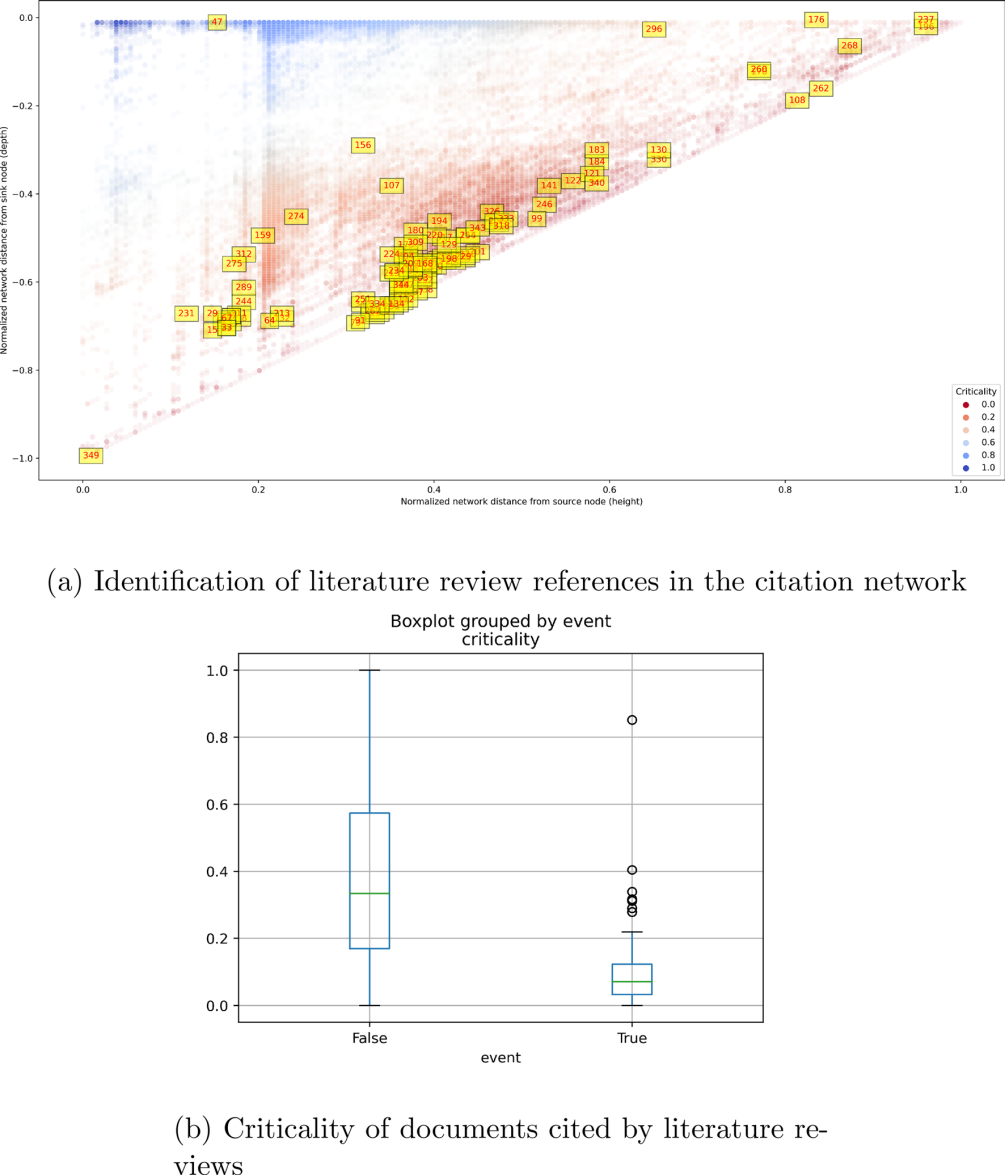
Critical innovation path is validated by literature reviews. Illustrative data from the Moderna Spikevax vaccine. (**a**) Depth: the maximum network distance from regulatory authorisation to any node; Height: the maximum network distance from the earliest innovation events. A low depth or high height represents proximity to therapeutic approval in the citation network and vice versa; critical innovation path (red nodes): composed of nodes whose height is approximately equal to depth meaning they are on the longest path of the network, approximating the importance of a node to the progression of the technology. A low distance from the longest path may indicate bottleneck to technological progress being overcome; a high distance may indicate the innovation event can happen at any time without obstructing technological progress. Documents in yellow are found in literature reviews by^34,35,51^; their presence validates our method. List of labelled documents are available at 10.6084/m9.figshare.22154030. (**b**) Event: inclusion of network documents in literature review articles identified *ex post*; true event: all documents within the network and identified in literature reviews; false events: all documents within the network and *not* identified in literature reviews. The box extends from the Q1 to Q3 quartile values of the data, with a line at the median (Q2). The whiskers extend from the edges of box to show the range of the data.

We validate the critical path by checking for documents that also appear in literature reviews published by subject-matter experts. Figure 5a shows the height versus depth diagrams (as described in Fig. 2) for the Moderna mRNA vaccine but with additional annotations showing 352 documents found in three literature reviews on mRNA vaccines^35,51,51^. We found that the critical path (the hypotenuses) are heavily populated by documents referenced in the literature reviews. Figure 5b shows that documents found both in the Moderna Spikevax vaccine network and literature review have lower median criticalities of 0.0710 [0.169,0.574] (where we give 25% and 75% in brackets) compared to documents found only in the former where the median is 0.0333 [0.169,0.574]. Kolmogorov–Smirnov tests indicate that criticalities of documents found in literature reviews is significantly different to that of documents not found in literature reviews (the p-values are always much less than 0.0001), validating the use of the critical path method to identify important innovation events.

## Discussion

When studying innovation, using a more complete citation network by integrating publication, patent, clinical trial, and regulatory data, allows this study to causally trace more intermediate steps between innovation inputs and an innovation outcome. Rather than regressing a limited set of variables, a citation network is a DAG, so this encodes the geometry of innovation order. Theoretically, the longest paths in a DAG represent the critical causal routes which show the bottlenecks that constrain innovation; it is the most complex route to achieve because it is composed of the largest amount of linear components which cannot be parallelised. We hypothesise that the longest paths in these multilayer citation networks, where order matters, are the critical paths of innovation.

The use of longest paths, not shortest paths (section F.2 in appendix), to analyse DAGs draws parallels in operations research, where the critical path method is used to schedule jobs in a project, such as the Manhattan Project^52–54^ or independent parts of a numerical simulation running on multiple processors. In this method, the DAG captures the dependency (the edges) of one job (one node) on another. An innovation network path length, considered as a scheduling DAG, is the sum of the time needed to complete each job on the path (so not simply the number of edges in the path). The aim is to find the “critical path” which is the path that sets the least time needed to complete the project. The critical path is set by the longest path in the scheduling DAG

Badiru^54^ defines three important aspects of the critical path method that we test using larger and more complex innovation datasets:

1. An activity is considered *critical* if changing the start or finish time of the activity will affect the overall project schedule.
2. The series of critical activities connecting the start and end points of a project is known as the *critical path*. Logically, the critical path “turns out to be the *longest path* in [a] network”.
3. A delay in any critical activity delays the entire project. Therefore, the “sum of durations for critical activities represents the *shortest possible time* to complete the project”.

This means:

*Critical schedule path* $\equiv$ *longest network path* $\equiv$ *shortest time path*

There is also a more formal mathematical basis for the use of longest paths in DAGs. In an innovation network, the longest path includes the most developmental steps of a final innovation, thus providing the most comprehensive evidence of the foundations of an emerging technology, while other paths, such as the shortest path, may overlook some important steps.

As an analogy for the longest path, imagine you are in a lift within a skyscraper. If you ride straight to the top floor, you will only see the CEO’s office, which could be your most direct route to a targeted endpoint. In a DAG, this is the shortest path; it is quick, but you bypass all other nodes. Now, think of the lift stopping at each floor along the way. You are introduced to every department, which is not “quick” but comprehensive in terms of collecting information. In DAG terms, this mirrors taking the longest path; you gain a comprehensive understanding of the whole network. Our dynamic representation of innovation as a DAG resembles the DAGs created within a Lorentzian space-time framework in the context of special relativity, where the passage of time is fundamental. For such DAGs embedded in Lorentzian space-times, it is found that the longest path is the closest approximation to the geodesics, which are the easiest routes for the matter (information) to follow^55–59^.

As seen in Fig. 2, innovation occurs both inside and outside the longest path. This paper does not suggest we do not need to innovate outside the longest path. Instead, the longest path offers a systematic and robust way to discern “critical” innovations, those lying close to the longest path and are pivotal for a specific technology’s progress, and “essential” innovations, those required for a final technology output, but have low criticality. Critical innovations often arise due to technical reasons, where discovering “X” is a prerequisite for inventing “Y”. Alternatively, they may stem from informational reasons, where understanding “X” highlights the value of funding “Y”, even if “Y” could have been developed earlier. Some documents may possess low criticality if they were created before the time when their context became relevant, were intended for other applications, or already existed for other reasons, and thus do not hold back technological emergence. This paper also does not suggest innovating along the longest path would remove all innovation frictions. However, assuming each innovation unit cannot be substituted, the innovation order will be identical regardless of other factors, such as funding increase. Thus, the longest path spots innovations that are possible now but which, if left till later, when their need is more obvious, will hold up technological emergence. Aligning more closely to the longest path in innovation programs offers the potential to avoid a “just too late” scenario when a “just in time” approach is possible.

One of the advantage of this study is that we do not focus on one longest path among the possible many, unlike other longest path-type methods. Equation 1 allows us to easily find all documents on any longest path; even better we can also look at documents that are close to the longest path, preventing falsely rejecting critical innovations and compensating for noise from imperfect citation data when we identify critical innovations. To verify the usefulness of the longest path in describing critical innovations, we prototyped two methods: (i) a reproducible way to construct multilayer citation network, thus representing basic and research (publications), invention (patents), development (clinical trials), and commercialisation (regulatory authorisations), and (ii) a simple way to quantify a document’s closeness to the longest path. These methods allow us to analyse events that turn out to be in the longest paths of eight vaccine citation networks. We were able to observe how basic discoveries in the lab accumulated and got absorbed by clinical researchers, who used these phenomenological observations to hypothesise what could work for a vaccine. Once a prototype vaccine product was available, the technological community further applied basic discoveries to optimise the product, which was eventually validated through clinical trials and approved for marketing.

As seen in Fig. 1, this paper provides the methodological foundation to define the types of edge in terms of the labels of the nodes at the end of the edge. This way of defining edges is useful in understanding innovation “translation”. The proposed method to quantify the criticality of innovation events opens opportunities for scientific understandings of technological change, particularly in: (1) comparing criticality patterns across industries and time spans and (2) attributing technological outcomes to events, entities, and policies^60^. Achieving this vision would propel the innovation community towards a “science of science policy”^61^.

Back in 1986, Derek Price^62^, who proposed Price’s model, imagined innovations as a cloud of gas bound to the laws of thermodynamics concerning such properties as the “volume of science” (quantity of science journals, papers, manpower, institutions, industrial output), “velocity distribution of [science]’s molecules” (academic citation and knowledge accumulation), and the “way in which [scientific] molecules interact with one another” (academic citation and knowledge concentration). The long-term dynamical relation between network height and innovation speed, shown in Fig. 3b, warrants further investigations about the factors at play in this relation, such as the formation of technology domains, number of suppliers and consumers, and number of nodes and citations. Future studies could also use Fig. 3b as a measurable interpretation of Utterback and Abernathy’s^63^ industry lifecycle model, which hypothesises that the “rates of” product and process innovation over time are convex and concave respectively; as well as the linear innovation model^16,17^, which prescribes that basic research, applied research, development, demonstration, and application be carried out by different sets of actors sequentially.

By assembling a list of innovation events in the observed technological past, we inform the ingredients needed in similar future technological programs. Using the longest DAG path as an innovation ruler, this proof-of-concept analysis demonstrated the possibility of measuring the rate of innovation, division of innovation labour, and qualify the impact of different stakeholders in the innovation process. By describing science and its funders more holistically, this study creates the opportunity for deeper learning about innovation.

### Supplementary Information

Supplementary Information.

## Data Availability

The data that support the findings of this study are available from Dimensions.ai, Lens.org, ClinicalTrials.gov, and the US Food and Drug Administration but restrictions apply to the availability of these data, which were used under license for the current study, and so are not publicly available. Data are however available from the authors upon reasonable request and with permission of the mentioned sources. De-identified network node and edge data used to support the main findings are deposited at https://doi.org/10.6084/m9.figshare.22155242 and https://doi.org/10.6084/m9.figshare.22154030.
