# Supplementary material for: Dynamics of technology emergence in innovation networks
Source: Sci Rep. 2024 Jan 16;14:1411. doi: 10.1038/s41598-023-50280-4 (PMC10791630; doi:10.1038/s41598-023-50280-4)
Supplement: Supplementary file 1 — Supplementary Information. [file 41598_2023_50280_MOESM1_ESM.pdf]

Supplementary Information for Dynamics of Technology Emergence in Innovation Networks

Martin Ho[1,2§*], Henry CW Price[3,4§], Tim S Evans[3,4‡], Eoin O'Sullivan[1,2‡]

1 Centre for Science Technology & Innovation Policy, University of Cambridge, Cambridge CB3 0HU, United Kingdom

2 Department of Engineering, University of Cambridge, Cambridge CB3 0HU, United Kingdom

3 Centre for Complexity Science, Imperial College London, London SW7 2AZ, United Kingdom

4 Theoretical Physics group, Department of Physics, Imperial College London, London SW7 2AZ, United Kingdom

§These authors contributed equally to this work.
‡These authors also contributed equally to this work.
* Corresponding author: wtmh3@eng.cam.ac.uk

A Formal definitions

Here we give the definitions of the network properties used in the main text using a formal language. The main aim is to arrive at a formal proof that nodes with zero criticality lie on a longest path in the DAG, Lemma A.4.

Definitions A.1. *Network/Graph, Nodes, Edges*
A NETWORK *(here synonymous with* GRAPH *)* $\mathcal{G} = (\mathcal{V}, \mathcal{E})$ *is a set of* NODES *(also known as vertices)* $\mathcal{V}$ *and a set of* EDGES $\mathcal{E} \subseteq \mathcal{V} \times \mathcal{V}$*. An edge is denoted* (u, v) *where* $u, v \in \mathcal{V}$*.*

Definitions A.2. *Layers*
A MULTILAYER NETWORK *is a graph where nodes are connected by different types of edges (for example, see section 4.2 in [1]). In our case, the* LAYERS *are defined by a partition of the nodes into different types* $\mathcal{V}_\alpha$ *so each layer contains nodes of only one type and each node exists on just one layer (a particular type of multilayer network). In our setting, we have an unweighted graph with four main types (Publication, Patent, Clinical trials, Regulatory approval) of nodes. The label of the node at each end of an edge, say* α *and* β*, leads to a partition of the edges into different types* $\mathcal{E}_{\alpha\beta}$ *(the definition of a multilayer network), that is* $\mathcal{E}_{\alpha\beta} \subseteq \mathcal{V}_\alpha \times \mathcal{V}_\beta$*.*

Definitions A.3. *Directed Graphs and Edges*
A DIRECTED NETWORK *has* DIRECTED EDGES *where the edge* (u, v) *is distinct from the edge* (v, u)*.*

Definitions A.4. *Predecessors and In-Degree*
The PREDECESSORS $N^-(v)$ *of a node* v *is the set of nodes which are connected by an edge* to v*, so* $\mathcal{N}^-(v) = \{u|(u, v) \in \mathcal{E}\}$
The IN-DEGREE $k_v^{(\text{in})}$ *of a node* v *is the number of incoming edges,* $k_v^{(\text{in})} = |\mathcal{N}^-(v)|$*.*

Definitions A.5. *Successors and Out-Degree*

The SUCCESSORS $N^{+}(v)$ *of a node* v *is the set of nodes which are connected by an edge* from v*, so* $\mathcal{N}^{+}(v) = \{w|(v,w) \in \mathcal{E}\}$*.*

The OUT-DEGREE $k_v^{(\mathrm{out})}$ *of a node* v *is the number of outgoing edges, so* $k_v^{(\mathrm{out})} = |\mathcal{N}^{+}(v)|$*.*

Definitions A.6. *Walk, Path, Cycles and Path Length*

A WALK *from node* u *to node* v*, denoted* $\mathcal{P}(u,v)$*, is a sequence of nodes starting at* u *and finishing with* v *which are connected sequentially by edges*

$$\mathcal{P}(u,v) = \{w_n | n = 0, \ldots, \ell, w_n \in \mathcal{V}, (w_n, w_{n+1}) \in \mathcal{E} \text{ for } 0 \leq n < \ell, w_0 = u, w_n = v\} . \quad \text{(A.1)}$$

The LENGTH OF A WALK *is the number of nodes minus one* $\ell = |\mathcal{P}| - 1$*.*

A PATH *is a walk where all the nodes are distinct, so* $w_n = w_m$ *iff* $m = n$ *in* (A.1)*.*

A CYCLE *is a walk where the first and last node in are identical,* $w_0 = w_\ell$ *in* (A.1)*, but all other nodes are distinct.*

The CONCATENATION OF TWO WALKS *is where a walk* $\mathcal{P}(u,v)$ *from* u *to* v *is combined with a walk* $\mathcal{P}(v,w)$ *from* v *to* w *to produce a walk* $\mathcal{P}(u,w)$ *from* u *to* w *via* v*. We simply extend the sequence of nodes in the first walk* $\mathcal{P}(u,v)$ *with the nodes in the second walk* $\mathcal{P}(v,w)$ *keeping the nodes in the same order and we only include the common end/start node* v *once. We denote this as* $\mathcal{P}(u,w) = \mathcal{P}(u,v) \cdot \mathcal{P}(v,w)$ *and formally we may define this as*

$$\begin{aligned} \mathcal{P}(u,v) \cdot \mathcal{P}(v,w) \;\; = \;\; & \{x_n | x_n = w_n \in \mathcal{P}(u,v),\ n = 0, \ldots, L_1; \\ & \qquad x_{n+L_1} = w_n \in \mathcal{P}(v,w),\ n = 0, \ldots, L_2\} , \\ & \qquad L_1 = |\mathcal{P}(u,v)| - 1 , \quad L_2 = |\mathcal{P}(v,w)| - 1 . \end{aligned} \quad \text{(A.2)}$$

Definitions A.7. *Directed Acyclic Graph — DAG, Sources and Sinks*

A Directed Acyclic Graph*, a* DAG*,* $\mathcal{D} = (\mathcal{V}, \mathcal{E})$ *is a network/graph with directed edges containing no cycles.*

A node s *with no incoming edges, that is* $\nexists\, u \in \mathcal{V}$ *s.t.* $(u,s) \in \mathcal{E}$*, is known as a* SOURCE NODE

A node t *with no outgoing edges, that is* $\nexists\, w \in \mathcal{V}$ *s.t.* $(t,w) \in \mathcal{E}$*, is known as a* SINK NODE

Definition A.8. *The Partial order of a DAG*

A DAG always defines a unique PARTIAL ORDER *on the set of nodes* $\mathcal{V}$ *in which we have a binary relation between two nodes, denoted* $u \prec v$*, if and only if there is a path from* u *to* v*.*

$$u \prec v \;\Leftrightarrow\; \exists\, \mathcal{P}(u,v) \;\; \forall\, u, v \in \mathcal{V} \quad \text{(A.3)}$$

Note that under our definition of a path, every node is part of a trivial path of length zero $\mathcal{P}(v,v) = \{v\}$ so this binary relation is reflexive, i.e. $v \prec v$, as required. Transitivity of a partial order comes from the fact that a concatenation of paths produces a path.

Definition A.9. *Distance between nodes*

The distance from node u *to node* v *in a DAG is defined to be the length of the longest path from* u *to* v*, while the distance is left undefined if there is no such path.*

$$d(u,v) = \max(|\mathcal{P}(u,v)| - 1 | u \prec v) . \quad \text{(A.4)}$$

This definition of a 'distance' in (A.4) is not sufficient for this function d to be a distance in the formal sense used in mathematics because (a) many pairs nodes in a DAG may not be connected and this distance is undefined for such pairs and (b) this definition is not symmetric

since if $d(u, v)$ is defined then $d(v, u)$ is not defined unless $u = v$. Note that both of these issues are easy to fix if a distance in the formal mathematical sense is required[1].

Note that there are often many paths between two nodes u and v with the same length. This includes the longest paths, those with length equal to $d(u, v)$.

Also note that many other distances functions can be defined for pairs of nodes on a DAG such as the length of the shortest path between two nodes. We will only use the distance defined here in terms of the length of longest path.

Definition A.10. *Reverse Triangle Identity*

The distance between two nodes satisfies the REVERSE TRIANGLE IDENTITY

$$d(u, w) \geq d(u, v) + d(v, w) \quad \text{if } u \prec v, v \prec w \,. \tag{A.5}$$

The reverse triangle identity has the opposite inequality from that found in the usual triangle inequality. The other difference is this identity does not apply for other permutations of the three sites u, v and w.

Transitivity of the partial order guarantees that $u \prec w$. This means that we can consider one of the longest paths from u to v, say $\mathcal{P}_L(u, v)$, whose length gives the value to $d(u, v)$. Likewise, we know we have a path $\mathcal{P}_L(v, w)$ which is a longest path from v to w of length $d(v, w)$. If we concatenate these two paths, we produce a path $\mathcal{P}_L(u, v) \cdot \mathcal{P}_L(v, w)$ from u to w which is of length $d(u, v) + d(v, w)$. The concatenated path is a path from u to w, but it need not be a longest path between these two, even though it is made by combining two longest paths. Since the distance (A.4) is set by the largest path length, it means the length of the concatenated path sets a lower bound on the distance from u to w. By definition, if there is another path from u to w and if such a path is longer than the concatenated path $\mathcal{P}_L(u, v) \cdot \mathcal{P}_L(v, w)$, then this alternative path will set the distance $d(u, w)$ and that will be larger than $d(u, v) + d(v, w)$. Hence, the simple properties of paths and the maximum function in (A.4) give us our reverse triangle identity.

Definitions A.11. *Height, Depth and Criticality*

The HEIGHT $h(v)$ *of a node* v *in a DAG* $\mathcal{D}$ *is the length of the longest path to that node from* any *node.*

$$h(v) = \max(|\mathcal{P}(u, v)| - 1 | u \prec v, u \in \mathcal{V}) \,. \tag{A.6}$$

The DEPTH $d(v)$ *of a node* v *in a DAG* $\mathcal{D}$ *is the length of the longest path from that node* v *to* any *node.*

$$d(v) = \max(|\mathcal{P}(v, w)| - 1 | v \prec w, w \in \mathcal{V}) \,. \tag{A.7}$$

The HEIGHT $h_{\max}$ *of a DAG* $\mathcal{D}$ *is the largest height of any node*

$$h_{\max} = \max(h(v) | v \in \mathcal{V}) \,. \tag{A.8}$$

The CRITICALITY $c(v)$ *of a node* v *in a DAG* $\mathcal{D}$ *is the height of the DAG minus the height and minus the depth of that node*

$$c(v) = h_{\max} - h(v) - d(v) \,. \tag{A.9}$$

The terminology 'height' and 'depth' are common when working with DAGs but 'criticality' is our own terminology for $c(v)$ in (A.9).

If the distance function d satisfies the properties of a formal mathematical distance then the height and depth are always defined and they will have a value of at least zero, $h(v), d(v) \geq 0$.

[1] For instance, consider $d'(u, v)$ where $d'(u, v) = d(u, v)$ of (A.4) when $u \prec v$. We then define $d'(v, u) = d(u, v)$ when $u \prec v$. Finally we set $d(u, v) = \infty$ whenever $u \not\prec v$ and $v \not\prec u$. This function d' is a formal mathematical distance function.

The only nodes with height zero are source nodes and the only nodes with depth zero are sink nodes.

With height and depth, we are effectively defining a distance between a node v and the 'beginning', some source node s, and the 'end' of our DAG, some sink node t. We can formalise this in the following lemmas, which lead to our key result on the bounds for criticality of a node and on the interpretation of zero criticality value for nodes in a DAG.

Lemma A.1 (Path leading to the height of a node). *The height of a node v is always the length of a longest path from some source node s to v.*

Proof. Suppose this were not true and the height of v is based on a path from some node u to v. If u is not a source node then there must be a node preceding u connected by an edge $(u', u) \in \mathcal{E}$. By definition $d(u', u) = 1$ and so by the reverse triangle identity (A.5) we know that $d(u', v) \geq 1 + d(u, v)$ so $d(u', v) > d(u, v)$. Thus, the node u does give the longest path to v and this node u does not define the height of node v. We have a contradiction and so deduce that the height of the path must use a node with no predecessors, i.e. a source node. □

Using the same type of argument used to prove Lemma A.1 we can quickly show the following lemma.

Lemma A.2 (Path leading to the depth of a node). *The depth $d(v)$ of a node v comes from the longest path from v to a sink node.*

We show this using the same arguments used in Lemma A.1 but now applied to paths from node v to some node w. If w has a successor, w', i.e. (w, w') is an edge, then node w is not involved in defining the depth.

Lemma A.3 (Path leading to the height of a node). *The height of a DAG, $h_{\max}$, comes from the length of a longest path from a source to a sink node.*

Proof. We use the same arguments as we did for the last two lemmas. The only paths that can not be extended at either end to produce longer paths are those running from a source node to a target node. Thus the nodes with the largest heights are the sink nodes. The height of the DAG comes from the largest of all heights (A.8), so this must run to a source node and, by Lemma A.1, run from a sink node. □

A couple of corollaries follow from this. First that the height of the DAG is the length of a longest path anywhere in the graph. Second that the height of the graph is also equal to the largest value of the depth which has to be for the depth of one (or more) of the source vertices.

Finally, we can put these ideas together to show the following lemma that is the basis for our analysis.

Lemma A.4 (Zero criticality nodes). *A node v with zero criticality, $c(v) = 0$, lies on a longest path in the DAG. Nodes v with positive criticality $c(v) > 0$ do not lie on a longest path of the DAG.*

Proof. Consider a node v with height $h(v)$ based on a longest path P_1 from source s to v (from Lemma A.1) and depth obtained from some longest path P_2 from v to sink node t (from Lemma A.2).

The path $P_1 \cdot P_2$ from source s via v to sink node t obtained by concatenating paths P_1 and P_2 has length $h(v) + d(v)$ by definition of concatenated paths in (A.2). This concatenated path $P_1 \cdot P_2$ need not be a longest path from s to t, which has length $d(s, t)$, but in that case, $P_1 \cdot P_2$ must be shorter than this longest path so $d(s, t) > h(v) + d(v)$. Equality, $d(s, t) = h(v) + d(v)$, can therefore only happen if the concatenated path $P_1 \cdot P_2$ is also a longest path from s to t.

If this longest path from source s to source t is one of the longest paths in the DAG, then $d(s,t)$ is the height $h_{\max}$ of the DAG. In this case we have $h_{\max} = d(s,t) = h(v) + d(v)$ and so $c(v) = 0$ which is the first of the lemma.

The converse of this statement, the second part of the lemma, follows from the following cases. Case (i) is where the concatenated path $P_1 \cdot P_2$ is a longest path from source s to source t but is *not* one of the longest paths in the DAG so $h_{\max} > d(s,t) \geq h(v) + d(v)$. Case (ii) is where the concatenated path $P_1 \cdot P_2$ is not a longest path from source s to source t so $d(s,t) > h(v) + d(v)$. In this second case, we know $h_{\max} \geq d(s,t)$ regardless of the nature of the concatenated path so again $h_{\max} > d(s,t) = h(v) + d(v)$. In either case, v is not on a longest path in the DAG and $c(t) > 0$ proving the second part of the lemma. $\square$

As a corollary of this lemma, we can see that the HEIGHT of the DAG $h(\mathcal{D})$ is also the largest possible value of the depth of any node.

B Methods in Detail

In this section, we look at how we move from raw data to produce the citation networks which encode the multiplicity of innovation phases. One important feature is our integration of multiple sources of data. Another key difference to earlier work is that the direction of time in a citation network is fundamental to our approach. We give a formal set of definitions in Appendix A.

B.1 Data

We create a multilayer citation network which is a directed acyclic graph (DAG) in order to observe innovation patterns and to test the relationship between critical scheduling events and documents on or close to the longest paths in the network, as discussed later in Section 4.

Vaccination confers long-lasting and protective immunity by presenting antigens of interest to elicit specific antibody production in recipients. Historically, vaccines present antigen through inactivated or attenuated version of whole or protein subunits of pathogens. Beyond efficacy, to prevent the spread of infectious agents, vaccines are administered to a large proportion of a population. Hence, vaccine must be safe and inexpensive. As a rapid countermeasure to such pathogenic outbreaks, other bottlenecks for vaccine platforms are manufacturability and ease of deployment. We outline the four vaccine platforms covered in this analysis and some technical events we expect to recover from the network in Table B.1. In Appendix C we give further details of the data sources used and the innovation events we expect.

Each network we create starts from a single document approving a particular vaccine, and this is the only source node in that DAG.

We obtain our data on clinical approvals from the US Food and Drug Administration (FDA), European Medicines Agency (EMA), and the UK Medicines and Healthcare products Regulatory Agency (MHRA). When a product is authorised by any of the three entities, we use the first authorised date to represent novelty and scan for all available references from all three agencies' authorisations [2, 3, 4, 5, 6, 7, 8, 9].

B.2 Innovation network

We start by defining the key properties of the innovation networks used in our work. Formally, a network (or graph) is a set of nodes, and pairs of nodes can be connected by an edge. In our networks, each node represents a single document which is one of four types: an innovation outcome represented by regulatory authorisation, a clinical trial, a patent, or an academic

Table B.1: Information on the vaccines analysed here. The number of nodes and edges are those present in the multilayer citation network created from a multi-step snowball sample starting from the vaccine approval document.

**Vaccine network**	**Technology platform**	**Disease targeted**	**Developer**	**Year first approved**	**Source node**	**Data source**
Spikevax	mRNA	COVID-19	Moderna	2020	[2]	[2, 10, 11]
Comirnaty			BioNTech	2020	[3]	[3, 12, 13]
Vaxzeria	Viral Vector		AstraZeneca	2020	[4]	[4, 14]
Zabdeno		Ebola	Janssen	2020	[5]	[5]
Dengvaxia	Live Attenuated	Dengue	Sanofi Pasteur	2019	[6]	[6, 15]
Imvanex		Smallpox	Bavarian Nordic	2013	[7]	[7, 16, 17]
Nuvaxovid	Subunit	COVID-19	Novavax	2022	[8]	[8, 18, 19]
Shingrix		Shingles	GSK	2017	[9]	[9, 20]

publication. So, our networks are examples of what are called MULTILAYER NETWORKS, for example see [1], as each type of node can be visualised as placed on a different LAYER, see Fig. 1. Our edges, written as (u, v), are citations from one node u to another node v so our networks are examples of CITATION NETWORKS. Note that edges in citation networks have a sense of direction as (u, v) represents an entry listing document v in the bibliography of a document u, not the other way round. So, citation networks are examples of what are known as directed networks.

Citation networks also have a sense of order since a document cannot cite a later document so for an edge (u, v), document v must have been published before[2] document u.

As a result, there should be no cycles (loops) in our networks. That is, if we move from one node to a neighbour, respecting the direction of the edge, and then repeat these steps as often as we want (this defines what is called a 'walk' in a network [1]), we will never return to the same node twice. Thus, our citation networks are examples of what are called DIRECTED ACYCLIC GRAPHS (DAG). The direction and the lack of cycles in a DAG are a direct result of a sense of order that is present in all DAGs. In a citation network, the order is the arrow-of-time implicit in a citation network. This order in a DAG leads to several special properties, which we exploit in our work.

In practice, we find that our data initially gives networks where 0.07% of all edges are part of a cycle, for example, due to authors citing each others' paper during journal submission or mislabelling. We always remove these cycles (as described below) to ensure the networks we analyse are always DAGs.

[2]Our data gives a single date for each document but in reality one can associate several different 'publication' dates: application and grant dates for patents, date first appeared online as opposed to the official publication date written in the text of a journal publication [21], etc. So, the data used to build a citation network can have edges that go from an earlier to a later document at least according to any single date we assign to each document, something seen in any work with citation networks such as [22]. To portray novelty consistently, we use the first published date for publications, priority date for patents, and start date for clinical trials.

B.3 Growing an innovation network

The networks we use all start from a single seed node, known as the SOURCE NODE, representing the regulatory marketing authorisation for one vaccine. This is because the regulatory decision represents the first time a therapeutic product is marketed and thus marks an innovation breakthrough. This regulatory authorisation node will be the newest node in each network we consider and so the only node in that network with no citations, that is, no incoming edges. This is the only node in our initial set of nodes denoted $\mathcal{V}(0)$.

In the second step, we scan the regulatory authorisation for any publications, clinical trials, and patents. We denote these documents as part of the set of nodes $\mathcal{V}(1)$ at a 'depth' of one from the source vertex. An edge is added from the source regulatory document to each of these document nodes at depth one.

In addition, since regulatory documents do not normally contain patent ids, we also locate precise patents p associated with vaccines through supplementary information on drug manufacturer inserts and websites. We add a node p and a link (f, p) from the regulatory document f to each associated patent.

Once we have all the patents p associated with the regulatory document f, directly and indirectly through drug manufacturer inserts and websites and through the Intervention sections of clinical trial documents, we finish by looking at PATENT FAMILIES. Each of the patents we have found is part of a patent family, something mentioned in the patent information, giving us further patents, say p_a where label a identifies a patent in the same family as p. However, we do not add new nodes for each of these patents p_a. We do find all references from any associated patent p_a to any further document, say d. However, all of these references are represented as links from the single p node to document d, a node in the second level set $\mathcal{V}(2)$, that is we add a link (p, d). In some sense, the patent nodes p at this first level represent all patents in the same family. We do not do this for patents at higher levels.

Another way we expand the patents in the early parts of our citation network is that we look at the clinical trials t in the regulatory document f, where there is already a link (f, t). We then search patent databases for therapeutic names recorded in the "Intervention" sections of each clinical trial document. New patents p_t found this way are also added as nodes at the next level, part of $\mathcal{V}(2)$, with links (t, p_t). We also perform the same search for documents cited by patents in the same patent family as p_t as noted above.

Thirdly, we perform snowball sampling. That is, at the ℓ-th step of sampling, we have a set of documents $\mathcal{V}(\ell)$ which form the nodes most recently added to our DAG. We start the process from the set of documents $\mathcal{V}(1)$, those one step away from the regulatory approval document. We follow the references in these $\mathcal{V}(\ell)$ documents to create new edges, say edge (v, r) from document $v \in \mathcal{V}(\ell)$ to a document r listed in the bibliography of v. If we have not encountered a document r so far, then we add r to the next set of documents to be considered, namely $\mathcal{V}(\ell + 1)$. This process produces an exponential growth in the number of documents, so we have to terminate this process at some stage. We do choose to do this after three steps because of computational limitations. This leaves us with vertices defined by the four distinct sets $\mathcal{V}(0)$, $\mathcal{V}(1)$, $\mathcal{V}(2)$ and $\mathcal{V}(3)$. We also have all the edges defined by the references of the documents in the first three sets. The final step of this part of our process is to look at the references given in the last set of documents found, $\mathcal{V}(3)$. If any document v in this last set of documents found refers to a document r we have already added to our network, then we add an edge (v, r). Should this reference be to a document r not currently in our data set, we do not add this document r as a new node to our network, and neither do we add an edge to r.

We limit the growth of the network to three iterations for two reasons: (i) the graphs would have grown exponentially in the first iteration of the algorithm, such that any further network growth will capture innovations so distant in the past that it would be unclear whether we can attribute them to the innovation outcome; and (ii) such a growth will result in an unmanageable

graph due to computational and data-sourcing limitations. At the end of the process, our network will have several nodes with no outgoing edges, and these nodes are known as SINK NODES.

In doing this snowball sampling, we have access to the citation data on three types of document: clinical trials from `ClinicalTrials.gov`; patents from `Lens.org` [23]; and publication data from `Dimensions.ai` [24]. The exhaustiveness of the first two data sources rests on the fact that all drugs and biological products conducted under regulatory investigational new drug application must be registered on, `ClinicalTrials.gov` and that the FDA maintains a database of all drugs and biological products it has approved. Similar requirements exist for the European Medicines Agency.

As with any data on citations, our results will be incomplete. References in the original documents may not be captured in our data sets for a number of reasons, such as errors in the original document, incorrect transcription from the primary source to our electronic sources for citations, or simply some documents are not in our databases such as press releases or preprints ('grey' literature). The legal framework behind vaccines means that our data on regulatory approval, clinical trials and patents is likely to be better than journal citations, but no data is perfect. We have not attempted to study the effect of errors in our data, rather relying on the large scale of our data to provide some statistical safety net.

Our process up to this point has provided a directed network where the nodes always have two additional labels. First, we record which of the four types of documents a node represents. The second node label gives a single date which we call the publication date: the priority date for patents, the start date for clinical trials, and the official publication date for an academic article. Additional node information about the funding of the research reported in any document is discussed later.

However, we also require that our network is acyclic. While in principle one document only ever refers to older documents, which guarantees an acyclic network, in practice there are always cycles in raw citation networks. These arise because documents are never created in a single moment of time. In practice, documents have a range of dates, from the first formal submission of a document (such as the application to hold a clinical trial, filing of a patent, depositing a paper on a preprint server) through to a final version of a document (the end of a clinical trial, the award of a patent or the physical publication date assigned to a journal article). For journal articles, there are many possible dates we could use [21] but they usually differ by the smallest amount, typically less than a year. For clinical trials and patents, the range of dates associated with these documents can be over several years. Across the eight networks studied, the mean and standard deviation of the number of cycles per edge was 0.0022 ± 0.0008. Our final step is to remove one edge from every cycle to produce a true directed acyclic graph[3].

Some basic statistics on the eight networks we create from our data are given in Table B.2.

B.4 Longest path in a citation network

A path in a network is a sequence of distinct nodes, $\{u_0, u_1, \ldots, u_\ell\}$, where each consecutive pair of nodes forms an edge so (u_i, u_{i+1}) is an edge, from u_i to u_{i+1} if the edges are directed as here. In our case, paths are always moving backwards in time, as each document in a path can only cite an older document as the next step on a path. We will define the LENGTH OF A PATH, ℓ, to be the number of edges in the path (one less than the number of nodes). In particular, we will focus on the *longest* paths, not on the shortest paths normally encountered

[3]In practice, we use the `find_cycle()` function in the `NetworkX` package [25]. This is used iteratively to remove all cycles. When a cycle is found, the first edge of the cycle is removed from the graph and the function is run again until no cycles can be found. Alternative approaches can be used to produce an acyclic graph [22].

Table B.2: Basic network properties of the eight vaccine networks.

Vaccine network	Nodes					Edges
	Publication	Patent	Clinical trials	Funders	Grants	
Spikevax	62,112	24,407	10	1,286	25,043	786,563
Comirnaty	37,383	8,127	76	1,289	18,744	340,161
Vaxzeria	58,210	32,367	5	1,274	21,528	648,877
Zabdeno	77,359	47,145	9	1,371	27,561	953,002
Dengvaxia	9,986	2,681	30	505	2,079	81,716
Imvanex	38,979	5,298	24	922	13,129	357,320
Nuvaxovid	13,855	1,348	4	924	7,547	104,182
Shingrix	12,987	6,993	22	753	6,288	174,881

in network science, e.g. as in [1]. It is one of the special properties of a DAG that the longest paths are typically of a reasonable length, making them useful measures. See Section 4 for a more detailed discussion of why we work with the longest path. We will define the DISTANCE between pairs of nodes in a DAG to be equal to the length of the longest path between two nodes.

A key assumption in our work is that the most important steps for an innovation lie on or close to the longest path in an innovation citation network. We argue that this is because knowledge is built up incrementally. Even when there are leaps in development, they are built on the success or failure of the most recent attempts to develop science. Our longest paths contain many documents that made a small contribution to the final vaccine but we suggest that all the key documents will be there. By way of contrast, had we used the shortest paths to study our innovation networks, the most widely used path in Network Science [1], the shortest paths do not contain cumulative information of knowledge inheritance. The shortest path would miss information because a document may cite important but old documents and so the shortest path will miss more recent critical developments, see Fig. 1. For a longer discussion of our choice and possible alternatives, including the differences between the longest path in a network and critical path in a schedule, see the discussion in Section 4.

In order to study the longest paths, it is convenient to define two standard properties of nodes in a DAG. The HEIGHT $h(v)$ of a node v is the maximum distance from the source node (the seed authorisation document) to the node v while the DEPTH $d(v)$ is the maximum distance from node v to any of the sink nodes. The height of the DAG $h_{\max}$ is equal to the largest possible value of the height, $h_{\max} = \max\{h(v)\}$. The height of a DAG is also always equal to the largest depth of any node, which in our case is the depth of the seed node, the regulatory approval node and the only source node in our DAGs.

It is important to note that because our distance is integer valued, there can be many longest paths between any two nodes, not just one. Further, while we argue that critical developments will lie on a longest path, this is not something we can prove rigorously and, in any case, we can expect data used to form our citation network to be imperfect. Therefore, it is extremely useful to be able to look at documents that are not on one of the longest paths to the source node but instead lie on a path from source to sink that is one or two steps shorter than the longest path in the DAG. That is we will also consider documents that are *close* to a longest path. To quantify what we mean by 'close' in this context, we define CRITICALITY $c(v)$ for a node v as:

$$c(v) = h_{\max} - h(v) - d(v). \tag{B.1}$$

Criticality c takes integer values between zero and the largest possible value of height or depth, the height of the DAG $h_{\max}$. Any node which lies on a longest path of the DAG will have zero criticality. Equally, nodes which lie on a path from the source node to a sink node which is

c steps shorter than the longest path of the DAG will have a criticality value of c. Thus, the criticality value of a node can be thought of as the distance of a node to one of the critical paths down which the key innovations flow. Applying (B.1) to Fig. 1, nodes a to i have a criticality of 0, indicating they are on the longest path, whereas node h has a criticality of 1.

In other words, for a given node in the innovation network, the node's height from the source node and depth from a sink node are uniquely defined. The novelty of our analysis is that we derive the longest path in the network by taking the criticality using height and depth. The criticality values $c(v)$ of nodes not only shows which nodes lie on longest paths (nodes with zero criticality) but also associated nodes lying on "near-longest paths" (small values of criticality). It is easy therefore for us to find other critical innovations which may have been missed by any method based on a single path, c.f. conventional main path analysis [26, 27] which always returns a single path (see Section F.3 for further discussion of main path analysis).

B.5 Measuring funder activity as a function of time

For each node, there is a possibility that grants and funders linked to the research are reported in the associated document. We also look for specific entities in the acknowledgements for increased coverage. On *Dimensions*, some publications, patents, and clinical trials are connected to grant nodes, providing additional details such as the value of the grant[4], associated funder, and funding period. When measuring the effect of funders, we look at nodes and their associated funders, either directly via document-funder edges or indirectly via document-grant-funder edges, at one citation step from the grant attached to that project.

This information on the relationship between documents recorded in our multilayer citation DAG and funders means that every node n can be associated with a subset of funders[5]. We can now look at the properties of those nodes linked to any one funder, such as height and depth, and use various summary statistics, such as the median document height, to understand the different roles played by different funders in the innovation process.

[4]However, we do not use monetary information in this study as we do not know how grants are split up by several publications, patents, or trials

[5]We could think of this as a new layer forming a bipartite network between document nodes and funder nodes. In our work, we only look at simple measures relating to funders, so such a network description of the funding landscape is unnecessary here. All the networks we discuss here are multilayer citation networks, no funding or grants are encoded in the network structures we analyse.

C Empirical data

In our context, the sources of our DAGs are the nodes representing the FDA approval on one of eight vaccines. We have chosen to work with vaccines produced from one of four different methods, and we outline these different approaches and associated vaccines in this section.

C.1 Viral vector (adenovirus vector) vaccine platform

Technical principle and bottlenecks. A VIRAL VECTOR VACCINE (VVV) is a relatively novel vaccine platform that uses virus to infect host cells with the genes of pathogens; the infected host cells then transcribe and translate the genes into antigens of the pathogen. Following vaccination, T cells and B cells respond against both to the viral vector itself and, more importantly, the antigen encoded viral vector. Viral vector vaccines can rapidly adapt from one pathogen to another because only the gene of interest needs to be exchanged. An ADENOVIRAL VECTOR VACCINE (AVV) is a subtype of the viral vector vaccines that exploits the high transduction efficiency and pervasive tropism of adenoviruses to facilitate the expression of target antigen. An adenoviral vector vaccine is produced by deleting the replication genes from an adenovirus serotype and inserting the genetic sequence of interest to the virus. This is followed by viral vector production in manufacturing cells and purification [28]. Historically, the efficacy of a viral vector vaccine has be challenged by hosts' immunity against the viral vector [29] and the surveyed vaccines should exhibit mechanism to circumvent this issue: finding rare or non-human adenovirus serotypes and vectorising adenoviruses from non-human primates [30, 31].

Data. As of October 2022, four viral vector vaccines have been cleared by the FDA (the US Food & Drugs Administration) for use in humans; we consider two of them which represent the first uses of adenovirus as vaccine vector: Zabdeno[6] (against Ebola, developed by Janssen, first authorised for use in 2020) [5] and Vaxzevria (COVID-19, AstraZeneca, 2020) [4].

C.2 Nucleic acid (mRNA) vaccine platform

Technical principle and bottlenecks. Both DNA and RNA can elicit immune response, but to date, only MRNA vaccines have been authorised by the FDA. Similar to adenoviral vector vaccines, nucleic vaccines are highly immunogenic, easily adopted, and readily manufactured compared to inactivated/attenuated vaccines. The two currently commercially available nucleic vaccines work by expressing antigens of interest via nucleoside-modified mRNA encapsulated in lipid nanoparticles (LNP) [32]. To arrive at the mRNA vaccines we have, innovators had to understand how mRNA elicits an immune response, how to control the amount of innate inflammatory reactions to therapeutic mRNA, how to deliver mRNA to transfect cells; the synthesis, purification, and buffering of mRNA [33, 34, 35, 32].

Data. Similar to adenoviral vector vaccines, only two mRNA vaccines are authorised by the FDA at the time of writing: Spikevax (COVID-19, Moderna, 2020) [2] and Comirnaty (COVID-19, BioNTech/Pfizer, 2020) [3].

C.3 Whole pathogen (attenuated) vaccine platform

Technical principle and bottlenecks. The very first vaccine was an example of a WHOLE PATHOGEN VACCINE (WPV) and the word "vaccine", from the Latin "vaccinus", comes from Jenner's use of cow ("vacca") pox to prevent smallpox. A typical whole pathogen vaccine

[6]Mvabea, the second dose of the Zabdeno/Mvabea regiment uses modified vaccinia Ankara (a poxvirus) as vector.

contain microbes that are live but attenuated, meaning they are weakened strains, or they are inactivated, meaning they are killed or altered to prevent replication. For instance, the vaccines behind the eradication of polio contain inactivated poliovirus. Although whole pathogen vaccines are a two-century-old innovation, pathogenic attenuation or inactivation does not readily guarantee a viable vaccine due to immunogenicity, safety, and yield issues [36]. This has led to newer vaccines to experiment with novel techniques such as the use of hydrogen peroxide and gamma irradiation as alternative means of inactivation [37, 38].

Data. Since whole pathogen vaccines are based on the oldest approach to vaccination, we use two examples as a baseline for comparisons with novel mRNA and viral vector vaccine platforms. We choose two whole pathogen vaccines that were recently cleared by the FDA: Dengvaxia (Dengue, Sanofi, 2019) [6] which is one of the first vaccines against dengue, and Imvanex (Smallpox, Bavarian Nordic, 2013; aka. Jynneos) [7] which is a third-generation smallpox vaccine that is being used to control monkeypox outbreak. Interestingly, the modified vaccinia Ankara used by Imvanex is the same virus that serves as a viral vector vaccine vector for the Mvabea vaccine discussed above.

C.4 Subunits (recombinant protein) vaccine platform

Technical principle and bottlenecks. Compared to a whole bacterium or virion, SUBUNIT VACCINES contain one or more isolated constituents of a microorganism to stimulate a more targeted immune response. Subtypes of subunit vaccines make use of protein subunits isolated and modified whole proteins or partial peptides from pathogens; toxoid inactivated pathogenic poisons; polysaccharides or glycoproteins to mimic glycoproteins on cell surface of pathogens; or chemical conjugation of low-affinity polysaccharides with a high affinity protein carriers to improve B-cell recognition of the polysaccharides. Usually, subunit vaccines elicit lower immunogenicity than WPV. Strategies to improve subunit vaccine effectiveness include the use of adjuvants, multiple dosage regiments, condon optimisation to improve yield, and amino acid substitution stabilise the introduced peptide chains [8].

Data. We use recombinant protein subunit vaccine, which has been widely available since the 1980s [39], as another baseline to compare with the novel mRNA and adenoviral vector vaccine platforms, and for similarity with the even more, established WPVs. Following the same logic, we choose two recently FDA-cleared subunit vaccines that both contain recombinant protein subunits and use adjuvants to enhance immune response: Nuvaxovid (COVID-19, Novavax, 2022) [40] and Shringrix (Shingles, GSK, 2017) [9].

D All figures

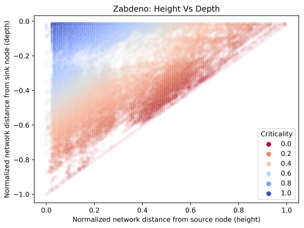

(a) Zabdeno, Ebola, Janssen, 2020, AVV

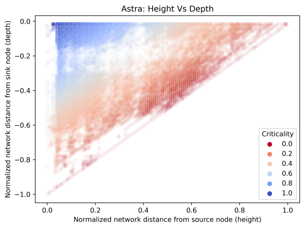

(b) Vaxzevria, COVID-19, AstraZeneca, 2020, AVV

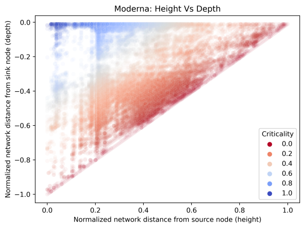

(c) Spikevax, COVID-19, Moderna, 2020, mRNA

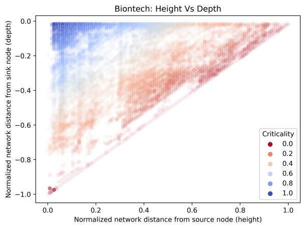

(d) Comirnaty, COVID-19, BioNTech/Pfizer, 2020, mRNA

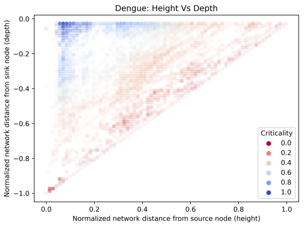

(e) Dengvaxia, Dengue, Sanofi, 2019, WPV

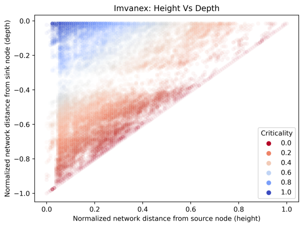

(f) Imvanex, Smallpox, Bavarian Nordic, 2013, WPV

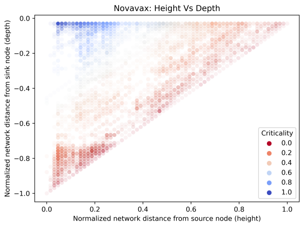

(g) Nuvaxovid, COVID-19, Novavax, 2022, subunits

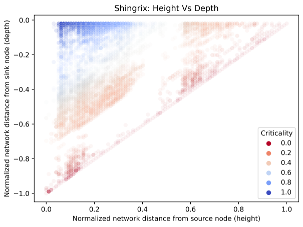

(h) Shringrix, Shingles, GSK, 2017, subunits

Figure D.1: **Depth as a function of height** with hypotenuse showing longest network path and critical innovation path.

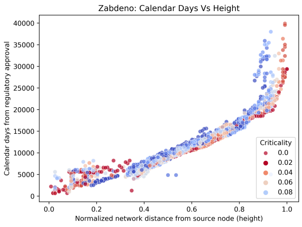

(a) Zabdeno, Ebola, Janssen, 2020, AVV

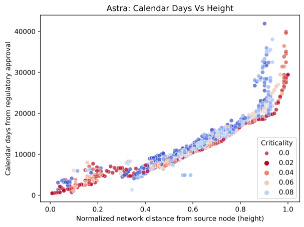

(b) Vaxzevria, COVID-19, AstraZeneca, 2020, AVV

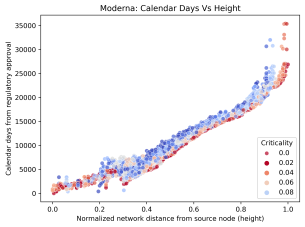

(c) Spikevax, COVID-19, Moderna, 2020, mRNA

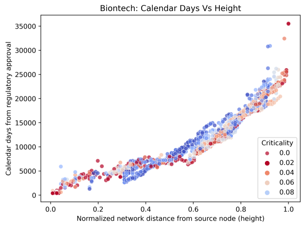

(d) Comirnaty, COVID-19, BioNTech/Pfizer, 2020, mRNA

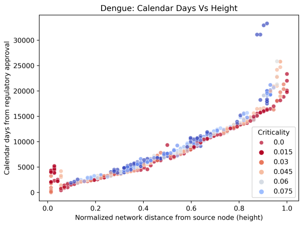

(e) Dengvaxia, Dengue, Sanofi, 2019, WPV

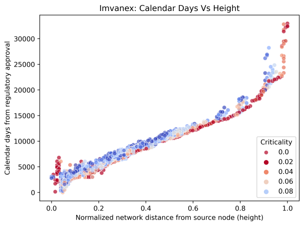

(f) Imvanex, Smallpox, Bavarian Nordic, 2013, WPV

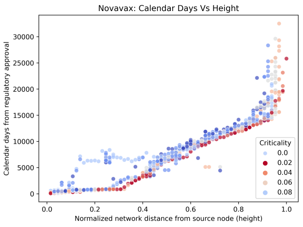

(g) Nuvaxovid, COVID-19, Novavax, 2022, subunits

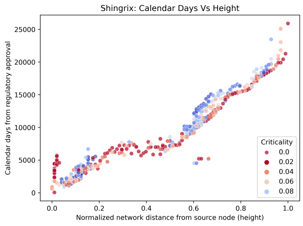

(h) Shringrix, Shingles, GSK, 2017, subunits

Figure D.2: **Calendar days as a function of network height.**

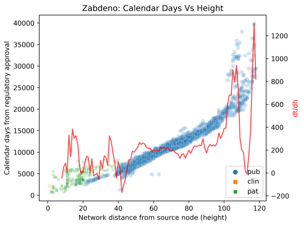

(a) Zabdeno, Ebola, Janssen, 2020, AVV

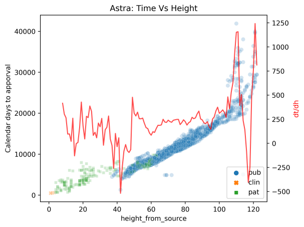

(b) Vaxzevria, COVID-19, AstraZeneca, 2020, AVV

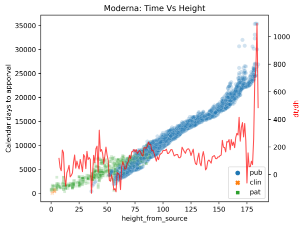

(c) Spikevax, COVID-19, Moderna, 2020, mRNA

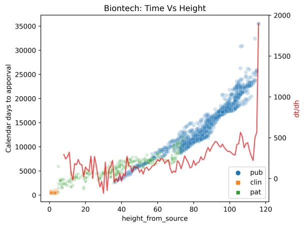

(d) Comirnaty, COVID-19, BioNTech/Pfizer, 2020, mRNA

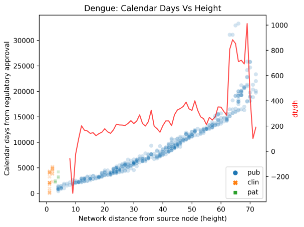

(e) Dengvaxia, Dengue, Sanofi, 2019, WPV

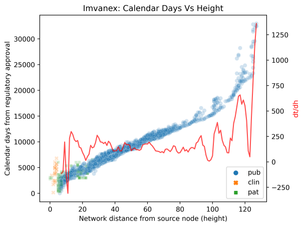

(f) Imvanex, Smallpox, Bavarian Nordic, 2013, WPV

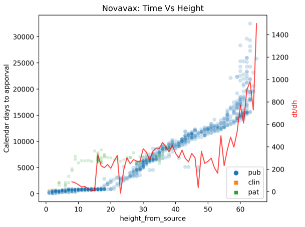

(g) Nuvaxovid, COVID-19, Novavax, 2022, subunits

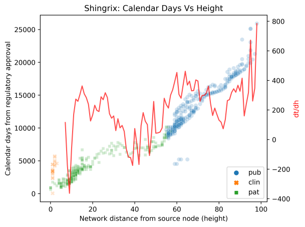

(h) Shringrix, Shingles, GSK, 2017, subunits

Figure D.3: **Calendar days per height as a function of height.**

—-

E Criticality of innovation funders is quantifiable via longest path

We take the definition of "critical" from operations research to mean an event that delays the global project schedule when locally delayed (see Section 4 for details). With the criticality information from the DAG, we also compute the criticality of funders, measured by the number of critical nodes funded by a particular entity divided by the total number of nodes funded by the entity in the DAG. We compare this performance metric with the citations received by documents the funder funds within the network.

Table E.1 shows that funders who fund research that, in turn, leads to large number of citations are not necessarily the funders of critical research. Entities that fund a high proportion of critical nodes are avid removers of innovation bottlenecks, who, in turn, allow progression along the technological trajectory. One caveat is that we do not know whether these critical funders deliberately removed innovation hurdles or unintentionally produced innovations that were applied to advance a technology by other entities. Another caveat is that some funders specialise in advancing basic science without thought of practical ends, while others specialise in translating innovation.

Table E.1: **Funding performance measured by critical path hit rate.** Illustrative data from Shingrix shingles subunits vaccine by GlaxoSmithKline. Critical path hit rate is the number of nodes funded on the critical innovation path divided by the number of nodes funded in the entire vaccine network (Section F.8 for details). Total citation counts the number of in-degrees of nodes funded by a funder within the DAG; citations can be by publications, patents, clinical trials, or regulatory authroisation within the DAG. Defense-related and commercial funders may not disclose certain critical innovations. The true critical path hit rate of these funders are likely to be higher than observed.

Funders on longest path	Funded nodes in entire network	Citations from funded nodes	Citations per funded node	Critical path nodes	Critical path hit rate (%)
Top 5 by critical path hit rate					
Defense Advanced Research Projects Agency	38	315	8	5	13.16
Swedish Research Council	59	246	4	4	6.78
GlaxoSmithKline (UK)	61	330	5	3	4.92
United States Public Health Service	217	1,139	5	6	2.76
National Institute of Allergy and Infectious Diseases	2,803	18,138	6	67	2.39
Top 5 by citations in DAG					
National Institute of Allergy and Infectious Diseases	2,803	18,138	6	67	2.39
National Cancer Institute	1,366	9,099	7	23	1.68
National Institute of General Medical Sciences	753	5,208	7	11	1.46
National Heart Lung and Blood Institute	451	3,193	7	8	1.77
National Institute of Diabetes and Digestive and Kidney Diseases	382	2,555	7	0	0.00

F Further methodological discussions

F.1 Concept of on causality in innovation systems

In short, this study approaches the concept of "causaltiy" in the sense that an earlier event *deterministically* traces to a later events. This study does not portray "causality" in the sense of the potential outcomes framework in econometrics, which *approximates* causal effects.

The concept of "causality" is central to many scientific inquiries but is approached differently across disciplines due to the varied nature of data and theoretical frameworks. Regression and natural experiment are prevailing method and experimental framework in economic studies of innovation systems.

In statistics, a correlation is observed when the regression results in a good data fit onto some line or curve. In econometrics and clinical statistics, a causation is further established if other variables that can affect the correlation are eliminated or corrected for.

The further quantification of cause-and-effect involves estimating the difference in trends between (i) what would have happened if there is an intervention (e.g. policy or medical intervention), and (ii) what would have happened if there is no intervention (i.e. the counterfactual). This is known as the Neyman-Rubin causal model [41]. In economic systems or policy, because only either (i) or (ii) can be the reality at each time point (e.g. there can either be a DARPA in US or not, you cannot have both scenarios at the same time), causal effects are estimated via the potential outcome reasoning, which involves "as-if" randomly assigning samples into (i) or (ii), so that the *average* treatment effect is the difference between the *average* outcome in the treatment group and the *average* outcome in the control group. This reasoning attempts to simulate the random assignment of economic entities as if patients in a clinical trial are randomly assigned into treatment and control groups in a hospital. This attempt is known as a "natural experiment".

Notable examples of natural experiments include difference-in-differences, which estimates the average treatment effect by comparing treatment and control groups, who would otherwise move in parallel without the treatment, in multiple time panels [42, 43]; and regression discontinuity, which exploits abrupt changes, cutoffs, or thresholds to "as if" randomly assign samples into treatment and control groups [44, 45, 46, 47]. For this reasoning to hold, an important assumption is that the treatment of any participant does not have an effect on other participants and that all confounding covariates are accounted for. Another validity requirement is the assumption that assignment is sufficiently "as if" random.

Based on this thinking, for example, it has been shown that winning SBIR grants from the Department of Energy *causes* a higher probability of receiving venture capital investments subsequently [48]. The regression approach is valid when a specific entity is being observed and that there is sufficient empirical data to cover the treatment, outcome, and confounding variables. In fact, regression, when properly done, has a reputation of being the gold standard of economic evidence. Nevertheless, due to the large number of entities and innovation phases and the permutations among them, regression appears unsuitable for analyzing complex technological and entity interactions.

This article argues "potential outcome reasoning" in natural experiments, commonly used in policy evaluation, is not the most appropriate in establishing causality in studying technological evolution. Rooted in clinical statistics, potential outcome reasoning estimates the causal effect of a treatment variable on an outcome variable by randomly assigning subjects into treatment and control groups so that, on average, as permitted by central limit theorem and law of large numbers, the treatment and control groups only differ by their treatment status and is independent of all other factors. In innovation systems, however, it is unlikely that (quasi-)random assignment can be achieved because ideas respect no barriers: There is no such thing as a "natural control" in innovation due to low marginal cost to adopt knowledge. Another

requirement of natural experiment is that treatment, outcome, and all confounding variables be accounted for unless there is an appropriate instrumental variable. Not only is it impractical to regress all relevant variables in an innovation system, often, the representativeness of innovation variables are sensitive to time. To illustrate, a drug in a Phase I trial is focused on toxicity, whereas the same drug at Phase III relies on efficacy variables. Problems about potential outcome reasoning are not unique to innovation economics: epidemiologists, who chiefly use randomized controlled trials, struggle with different states of a same variable, the specificity of variables (e.g. what variable can exhaustively denote innovation?), the context dependence of causality, and using different types of evidence to arrive at one overall verdict [49].

Interestingly, graph theory is used alongside, rather than as an alternative to, randomized controlled trials in epidemiology [50]. However, epidemiologists' use of networks is limited to non-parametric visual representations of variables in randomized controlled trials and *a priori* exploration of causal variables [51, 52]. Citation networks are a prime example of networks being analytically applied to causally order innovation events [53, 54]. Their ability to support causal inference is, nevertheless, marred by incomplete data. This is firstly because citation networks typically rely on one type of data, either patents only or journal publications only, meaning not all technological maturities are represented. Secondly, citation datasets are typically generated using keyword searches only [55, 56]. Searching for patents using keyword search, for example, "biofuel", would not result in a citation network that captures early scientific advances, in, for example, genetic engineering because the future applications of these advances were unknown. Thirdly, contrary to natural experiments that are restrictive in the dataset being used, it is often a challenge to delimit a specific dataset to construct a citation network. For instance, some citation network analyses can cluster millions of patents ever filed in a country by patent classification codes [57, 54].

This article is of the view that when sufficient data is available, the network view is a step up from regression approaches as the former makes less assumptions about perfectly rational and representative agents, random assignments, observing the averaged overall at equilibrium, homogenous interactions, closed systems, and static outcomes [58, 59]. In addition to relaxing the assumptions of neoclassical economics, the complex network approach retains information of intermediaries between inputs and outputs in a system to allow deterministic computation of causality (Fig. F.1), opening the doors to more realistic interpretation of innovation trajectories. In contrast to artificially assigning or approximating treatment, outcome, and control variables, the citations publications, patents, clinical trials are present in the data. These considerations reveal the philosophical position of this article.

F.2 Longest path vs shortest path

One of the most useful measures in network science is the length of the *shortest* path between two nodes as this is used in numerous situations as the distance between two nodes[7]. In many cases, these shortest paths are of practical relevance as we often look for the quickest, shortest route between two objects (nodes) in a network. For instance, in a social network, where people are nodes and edges are the connections between friends, the shortest path often represents the quickest way to get information between people. It is the basis for the popular idea of the six degrees of separation [1]. As a result, this shortest-path measure of distance between nodes is the basis for many other fundamental measures in network science, such as centrality measures [1].

In most networks, the longest path between two nodes has little practical relevance. For

[7]The length of the shortest path between two nodes in a network satisfies all the criteria is what is formally defined as a 'distance' function in mathematics. Indeed, it also satisfies the mathematical criteria to be a 'metric' and the shortest paths are therefore 'geodesics'.

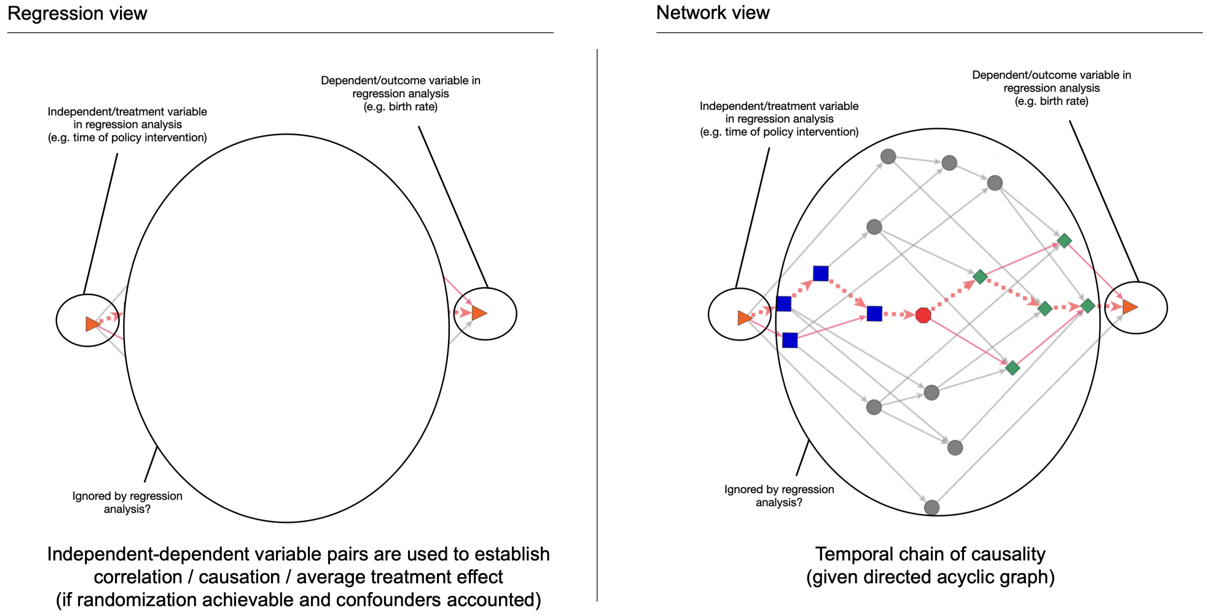

Figure F.1: **Regression versus network view of policy attribution.** Author's own analysis.

instance, in a social network, the longest path between two people will usually be a path visiting almost everyone in the network once, and we can think of no practical use for the distance of such a path in that context[8]. However, the order encoded in a DAG means that the longest path between two nodes in such networks is not especially long; the length of the longest path in a DAG is rarely similar to the number of edges, as it is in the social network example above. So, now the question is, which path should we use when analysing the flow of information in our multilayer innovation citation networks, the shortest or the longest paths?

At a qualitative level, we can see that the longest path is likely to be more interesting for citation networks. The oldest papers we cite in our bibliography here are over sixty years old. In preparing this paper, it is likely that we did not learn much of direct relevance to the current paper by reading such papers. Such old papers are classics, but their influence is indirect, felt in our present work via more recent publications which apply these classic concepts in a modern context with modern terminology and notation. Conversely, it seems likely that the most recent papers give a much more powerful stimulus to authors.

Most papers in the bibliography of a journal article are recent, and the time difference between the citing document and the documents in one reference list decreases exponentially when the age difference is a couple of years or more, [60, 61, 62, 63]. Further support comes from studies which show that the text for over 70% of references in the bibliography of a journal article may have been copied from the publications of intermediate age suggesting that a large fraction of these older articles are not read when a new article [64, 65, 66, 22]. That is consistent with the idea that the ideas in these older texts were learnt from intermediary texts. In the case of innovation, the longest path embeds a chronological sequence of key technological advancements. The longest paths in a DAG embed the chronological and causal sequence of

[8]Paths which visit every node are sometimes of interest, e.g. in the classic travelling salesman problem. However, in such cases the length of the paths is defined differently, in terms of the length of time to travel the network, the sums of the travel times associated with each edge. In such cases, there are many paths passing through all nodes, and the problem is still to find the shortest of these paths when distance is measured in terms of the sum of the time taken to travel each link in the path. So, this is still searching for the shortest paths out of a set of options, but using a different measure for distance from the one we are discussing at this point in the text.

key scientific and technological advances contributing to a technological outcome.

F.3 Main Path Analysis

Here we will give a brief summary of "main path analysis". This was proposed by Hummon and Doreian [67] and, with variations, has been implemented in some popular analysis packages, for example see [26].

Main path analysis starts by looking at all possible paths between a specified set of nodes, a set which varies between various implementations of main path analysis. The number of these paths which pass through a given edge is used to assign the "edge weight" of that edge, i.e. a value assigned to that edge. Then the length of a path is defined to be the sum of the edges weights traversed in that path. Finally, the "main path" is defined using a greedy algorithm to find paths of high length as defined using the edge weights. That is to construct a main path, the current main path is extended by adding one more node to the end of the path such that the length increases by the largest amount. The main path will end when it reaches a sink node, a node with no outgoing edges.

The first main path analysis described the scientific advances that eventually led to the discovery of the DNA structure by Watsons and Crick [67]. Main path analysis is later applied to other networks of academic publications [68, 69, 70, 71, 72, 73, 74, 75, 76, 77, 78] and, more recently, patents [70, 79, 80, 81, 82, 83, 84, 27, 85, 86]. Case studies of main path analysis span from understanding the emergence of engineered products such as battery, nanotubes, automobiles, and semiconductors; to academic theories such as bioinformatics, social network analysis, absorptive capacity, Hirsch index, and peer review. While popularity of main path analysis is one measure of a successful method, this popularity could be due to other factors such as the easy access to numerical implementations in widely used packages such as `pajek` [26]. Most of these case studies employ one of the four "out-of-the-box" indices developed by Hummon and Doreian [67] and Batagelj [26] with the objective to reduce the number of nodes in a citation network to a single chain of events to enable qualitative interpretation. However, these studies do not consider the longest path beyond a simplification device.

F.4 Edge weighting

We choose to assign a weighting of 1 to all edges because we assume each edge in the citation network, including in the longest path, represents a minimum viable increment of novelty. A weight of 1 allows equivalence in increments of innovation. We believe this assumption is valid because being published in a journal, accepted as a patent, approved to run a clinical trial, or authorised to market a therapeutic represents a minimum normalised threshold of originality from peer-review[9]. i.e a group would not be able to publish any earlier and would not delay publication as they would seek to publish an increment as soon as possible. Conversely, any reweighting, such as in main path analysis, impedes interpretability. Future works can use funding amount as weight if data becomes more complete; whereas we do not recommend using time as edge weights because time is already implied in network height and an edge that consumes a long duration of time does not mean it is more novel.

F.5 Citation behaviours

When studying citation networks, it is important to note that citation practices vary. Different types of document may have different goals, and publishers set their own constraints on the

[9]Anecdotally also known as the LPI or Least Publishable Unit `https://en.wikipedia.org/wiki/Least_publishable_unit`

bibliographies. The citation tradition in various fields can be very different, while individual authors add another source of variability. For instance, patent applicants need to strike the balance between minimising citations to demonstrate novelty and citing enough to not infringe prior arts [87, 88]. Based on fig. 3, we can believe privately-funded publications are likelier to have end-uses in mind and may bias citations towards applied research; whereas publicly-funded publications may be preoccupied with phenomenological questions. In addition, funders enforce grant acknowledgements in publications and patents differently. For example, the US Bayh-Dole Act requires that all recipients of federal research funds report to the funding agency any patent they file and acknowledge on patent documents the existence of federal funding, while many other countries do not have similar requirements. The different citation behaviours are likely more pronounced in the multilayer citation network we use as it assumes publications, patents, clinical trials, and regulatory approvals cite in the same way.

F.6 Patent family

A patent family is a collection of patent applications covering the same or similar technical content. Patent families usually arise from a single invention being filed in multiple countries ("simple patent family") and when an applicant files new applications for similar existing technical contents ("extended patent family"). Section F.7 below explains the importance of considering patent families in our network.

F.7 Patent prosecution

Patent application often spans several years. Four key dates in chronological order are:

1. Priority date: date used to establish the novelty of an invention
2. Filing date: when a patent application is first filed at a patent office
3. Publication date: when a patent application is published
4. Grant date: when a patent office grants a patent

Patent prosecution is the interaction among patent applicants, patent offices including examiners, and other interested parties. Patent prosecution usually spans between (2) filing date and (4) grant date, but can extend after grant if there is opposition, corrections, or other post-grant proceedings.

Due to patent prosecution, the bibliography of almost every patent is updated with new references. Almost every patent gets citations added during their prosecution time. These can be added by the examiner, by the applicant, assignee, or the inventor. What occurs less frequently is for citations to be added after grant. These usually happen for more limited reasons, e.g. post grant opposition, corrections, reissues, etc.

We use the initial patent submission date as our patent publication date. A year or two into the patent process, a recent paper can be added to the application, one that was published after the patent was submitted. As a result a patent may cite forward in time as well as the logically acceptable backwards in time. We could use the patent award date as our patent publication date which would solve the problem with the example just given. However, now we run into problems with documents that cite a patent that is not yet approved yet is a critical part of the innovation process. This illustrates why our using the height of a node in our citation network can be a more consistent record of the logical order in the innovation process compared to calendar time. We also address this issue by considering patent families rather than single patents when possible to capture references added to a patent during patent prosecution.

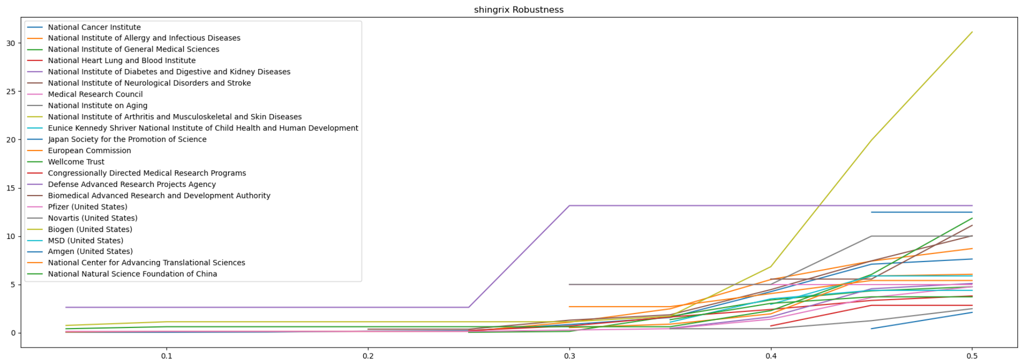

Figure F.2: **Number of critical path nodes funded by funders as a function of criticality threshold.** Illustrative data from Shingrix vaccine. x-axis: critical path threshold, y-axis: number of critical path documents for funders.

F.8 Critical path hit rate

The criticality of a given node is clearly defined in this paper by equation (B.1). Critical innovation path, on the other hand, depends on a threshold – nodes with a criticality below an arbitrary value would be considered residing on the critical innovation path. To provide a fair comparison across funders and across vaccines in Table E.1, we define the critical path as nodes whose criticality is below the maximum height in a DAG multiplied by a criticality threshold x. To determine the value of x, we conducted a robustness check (Fig. F.2) to determine that the criticality threshold for the Shingrix network would be 0.35 as this is when most funders become present on the critical path. In Table E.1, we only include funders who funded more than one node on the critical path and more than ten nodes in the entire network for meaningful comparison.

F.9 Network density

The density of nodes reflects ambiguity in the networks' local and global order. Fig. F.3 shows the citation networks are densest at low height and sparsest at high height. The latter is due to dangling nodes, potentially due to incomplete citation data in early years, meaning these regions are sensitive to change. On the other hand, observations drawn from other heights are more stable.

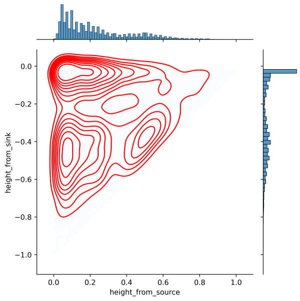

(a) Zabdeno, Ebola, Janssen, 2020, AVV

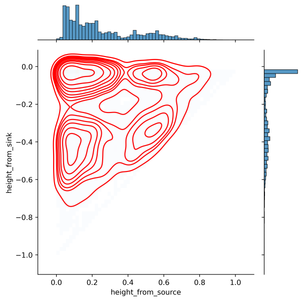

(b) Vaxzevria, COVID-19, AstraZeneca, 2020, AVV

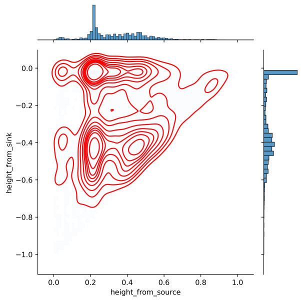

(c) Spikevax, COVID-19, Moderna, 2020, mRNA

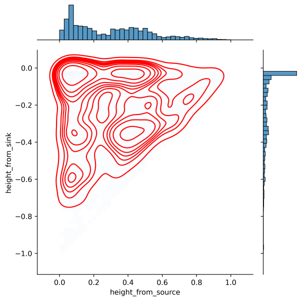

(d) Comirnaty, COVID-19, BioNTech/Pfizer, 2020, mRNA

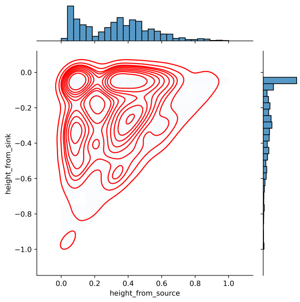

(e) Dengvaxia, Dengue, Sanofi, 2019, WPV

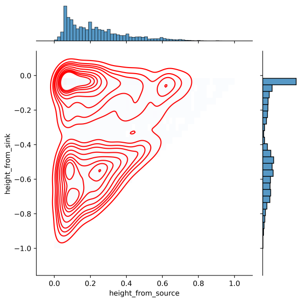

(f) Imvanex, Smallpox, Bavarian Nordic, 2013, WPV

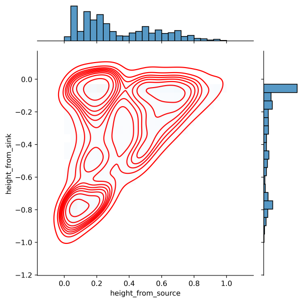

(g) Nuvaxovid, COVID-19, Novavax, 2022, subunits

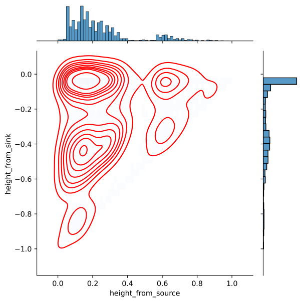

(h) Shringrix, Shingles, GSK, 2017, subunits

Figure F.3: **Kernel density estimation of height and depth.**

References

[1] M. Coscia, *The Atlas for the Aspiring Network Scientist.* Michele Coscia, 2021.

[2] Food and Drug Administration, "Moderna covid-19 vaccine eua letter of authorization," report, US Food and Drug Administration, 2020.

[3] Food and Drug Administration, "Emergency use authorization (eua) for an unapproved product review memorandum: Pfizer-biontech covid-19 vaccine/ bnt162b2," report, US Food and Drug Administration, 2020.

[4] Medicines and Healthcare products Regulatory Agency, "Public assessment report: National procedure: Vaxzevria," report, UK Medicines and Healthcare products Regulatory Agency, 2021.

[5] European Medicines Agency, "Assessment report: Zabdeno," report, Committee for Medicinal Products for Human Use (CHMP), 2020.

[6] Food and Drug Administration, "Approval letter - dengvaxia," report, US Food and Drug Administration, 2019.

[7] Food and Drug Administration, "September 24, 2019 approval letter- jynneos," report, US Food and Drug Administration, 2019.

[8] European Medicines Agency, "Nuvaxovid: Epar - public assessment report," report, European Medicines Agency, 2021.

[9] Food and Drug Administration, "October-20 2017, approval-letter, shingrix," report, BL 125614/0, 2017.

[10] European Medicines Agency, "Assessment report: Covid-19 vaccine moderna," report, European Medicines Agency, 2021.

[11] Medicines and Healthcare products Regulatory Agency, "Regulatory approval of spikevax (formerly covid-19 vaccine moderna)," report, UK Medicines and Healthcare products Regulatory Agency, 2021.

[12] European Medicines Agency, "Assessment report: Comirnaty," report, European Medicines Agency, 2020.

[13] Medicines and Healthcare products Regulatory Agency, "Vaccine bnt162b2 – conditions of authorisation under regulation 174," report, UK Medicines and Healthcare products Regulatory Agency, 2020.

[14] European Medicines Agency, "Assessment report: Covid-19 vaccine astrazeneca," report, European Medicines Agency, 2021.

[15] European Medicines Agency, "Assessment report: Dengvaxia," report, European Medicines Agency, 2018.

[16] European Medicines Agency, "Assessment report: Imvanex," report, European Medicines Agency, 2013.

[17] Medicines and Healthcare products Regulatory Agency, "Summary of product characteristics: Imv anex suspension for injection," report, UK Medicines and Healthcare products Regulatory Agency, 2021.

[18] FDA, "Emergency use authorization (eua): Novavax covid-19 vaccine, adjuvanted," report, US Food and Drug Administration, 2022.

[19] Medicines and Healthcare products Regulatory Agency, "Regulatory approval of covid-19 vaccine nuvaxovid," report, UK Medicines and Healthcare products Regulatory Agency, 2022.

[20] European Medicines Agency, "Assessment report: Shingrix," report, European Medicines Agency, 2018.

[21] S. Haustein, T. D. Bowman, and R. Costas, "When is an article actually published? an analysis of online availability, publication, and indexation dates," in *Proceedings of the 15th International Society of Scientometrics and Informetrics Conference*, pp. 1170–1179, 2015.

[22] J. R. Clough, J. Gollings, T. V. Loach, and T. S. Evans, "Transitive reduction of citation networks," *Journal of Complex Networks*, vol. 3, pp. 189–203, 2015.

[23] O. A. Jefferson, D. Koellhofer, B. Warren, and R. Jefferson, "The lens metarecord and lensid: An open identifier system for aggregated metadata and versioning of knowledge artefacts," *LIS Scholarship Archive Works*, 2019.

[24] D. W. Hook, S. J. Porter, and C. Herzog, "Dimensions: Building context for search and evaluation," *Frontiers in Research Metrics and Analytics*, vol. 3, 2018.

[25] A. Hagberg, P. Swart, and D. S Chult, "Exploring network structure, dynamics, and function using networkx," tech. rep., Los Alamos National Lab.(LANL), Los Alamos, NM (United States), 2008.

[26] V. Batagelj, "Efficient algorithms for citation network analysis," *arXiv preprint cs/0309023*, 2003.

[27] H. Park and C. L. Magee, "Tracing technological development trajectories: A genetic knowledge persistence-based main path approach," *PloS one*, vol. 12, no. 1, p. e0170895, 2017.

[28] C. Jacob-Dolan and D. H. Barouch, "Covid-19 vaccines: Adenoviral vectors," *Annual Review of Medicine*, vol. 73, no. 1, pp. 41–54, 2022.

[29] M. Saxena, T. T. H. Van, F. J. Baird, P. J. Coloe, and P. M. Smooker, "Pre-existing immunity against vaccine vectors–friend or foe?," *Microbiology (Reading)*, vol. 159, no. Pt 1, pp. 1–11, 2013. 1465-2080 Saxena, Manvendra Van, Thi Thu Hao Baird, Fiona J Coloe, Peter J Smooker, Peter M Journal Article Review 2012/11/24 Microbiology (Reading). 2013 Jan;159(Pt 1):1-11. doi: 10.1099/mic.0.049601-0. Epub 2012 Nov 22.

[30] P. Abbink, A. A. C. Lemckert, B. A. Ewald, D. M. Lynch, M. Denholtz, S. Smits, L. Holterman, I. Damen, R. Vogels, A. R. Thorner, K. L. O'Brien, A. Carville, K. G. Mansfield, J. Goudsmit, M. J. E. Havenga, and D. H. Barouch, "Comparative seroprevalence and immunogenicity of six rare serotype recombinant adenovirus vaccine vectors from subgroups b and d," *Journal of Virology*, vol. 81, no. 9, pp. 4654–4663, 2007.

[31] M. D. J. Dicks, A. J. Spencer, N. J. Edwards, G. Wadell, K. Bojang, S. C. Gilbert, A. V. S. Hill, and M. G. Cottingham, "A novel chimpanzee adenovirus vector with low human seroprevalence: Improved systems for vector derivation and comparative immunogenicity," *PLOS ONE*, vol. 7, no. 7, p. e40385, 2012.

[32] M. J. Hogan and N. Pardi, "mrna vaccines in the covid-19 pandemic and beyond," *Annual Review of Medicine*, vol. 73, no. 1, pp. 17–39, 2022.

[33] N. Pardi, M. J. Hogan, F. W. Porter, and D. Weissman, "mrna vaccines a new era in vaccinology," *Nature Reviews Drug Discovery*, vol. 17, no. 4, pp. 261–279, 2018.

[34] N. Chaudhary, D. Weissman, and K. A. Whitehead, "mrna vaccines for infectious diseases: principles, delivery and clinical translation," *Nature Reviews Drug Discovery*, vol. 20, no. 11, pp. 817–838, 2021.

[35] E. Dolgin, "The tangled history of mrna vaccines," *Nature*, vol. 597, no. 7876, pp. 318–324, 2021.

[36] B. Sanders, M. Koldijk, and H. Schuitemaker, "Inactivated viral vaccines," in *Vaccine Analysis: Strategies, Principles, and Control* (B. K. Nunnally, V. E. Turula, and R. D. Sitrin, eds.), pp. 45–80, Berlin, Heidelberg: Springer Berlin Heidelberg, 2015.

[37] M. Alsharifi and A. Müllbacher, "The gamma-irradiated influenza vaccine and the prospect of producing safe vaccines in general," *Immunol Cell Biol*, vol. 88, no. 2, pp. 103–4, 2010. 1440-1711 Alsharifi, Mohammed Müllbacher, Arno Letter United States 2009/10/28 Immunol Cell Biol. 2010 Feb;88(2):103-4. doi: 10.1038/icb.2009.81. Epub 2009 Oct 27.

[38] I. J. Amanna, H.-P. Raué, and M. K. Slifka, "Development of a new hydrogen peroxide–based vaccine platform," *Nature Medicine*, vol. 18, no. 6, pp. 974–979, 2012.

[39] P. J. Hotez and M. E. Bottazzi, "Whole inactivated virus and protein-based covid-19 vaccines," *Annual Review of Medicine*, vol. 73, no. 1, pp. 55–64, 2022.

[40] European Medicines Agency, "Chmp assessment report: Nuvaxovid," report, Committee for Medicinal Products for Human Use (CHMP), 2022.

[41] P. W. Holland, "Statistics and causal inference," *Journal of the American Statistical Association*, vol. 81, no. 396, pp. 945–960, 1986. doi: 10.1080/01621459.1986.10478354.

[42] R. Jensen, "The digital provide: Information (technology), market performance, and welfare in the south indian fisheries sector," *The quarterly journal of economics*, vol. 122, no. 3, pp. 879–924, 2007.

[43] G. Richardson and W. Troost, "Monetary intervention mitigated banking panics during the great depression: Quasi-experimental evidence from a federal reserve district border, 1929–1933," *Journal of Political Economy*, vol. 117, no. 6, pp. 1031–1073, 2009.

[44] T. D. Cook, ""waiting for life to arrive": A history of the regression-discontinuity design in psychology, statistics and economics," *Journal of Econometrics*, vol. 142, no. 2, pp. 636–654, 2008.

[45] C. Carpenter and C. Dobkin, "The effect of alcohol consumption on mortality: Regression discontinuity evidence from the minimum drinking age," *Am Econ J Appl Econ*, vol. 1, no. 1, pp. 164–182, 2009. 1945-7790 Carpenter, Christopher Dobkin, Carlos R01 AA017302/AA/NIAAA NIH HHS/United States R01 AA017302-01/AA/NIAAA NIH HHS/United States R01 AA017302-02/AA/NIAAA NIH HHS/United States Journal Article 2009/01/01 Am Econ J Appl Econ. 2009 Jan 1;1(1):164-182. doi: 10.1257/app.1.1.164.

[46] P. Bharadwaj, K. V. Løken, and C. Neilson, "Early life health interventions and academic achievement," *American Economic Review*, vol. 103, no. 5, pp. 1862–91, 2013.

[47] G. de Blasio, D. Fantino, and G. Pellegrini, "Evaluating the impact of innovation incentives: evidence from an unexpected shortage of funds," *Industrial and Corporate Change*, vol. 24, no. 6, pp. 1285–1314, 2014.

[48] S. T. Howell, "Financing innovation: Evidence from r&d grants," *American Economic Review*, vol. 107, no. 4, pp. 1136–64, 2017.

[49] J. P. Vandenbroucke, A. Broadbent, and N. Pearce, "Causality and causal inference in epidemiology: the need for a pluralistic approach," *International Journal of Epidemiology*, vol. 45, no. 6, pp. 1776–1786, 2016.

[50] J. Pearl, "Causal diagrams for empirical research," *Biometrika*, vol. 82, no. 4, pp. 669–688, 1995.

[51] T. C. Williams, C. C. Bach, N. B. Matthiesen, T. B. Henriksen, and L. Gagliardi, "Directed acyclic graphs: a tool for causal studies in paediatrics," *Pediatric Research*, vol. 84, no. 4, pp. 487–493, 2018.

[52] M. Piccininni, S. Konigorski, J. L. Rohmann, and T. Kurth, "Directed acyclic graphs and causal thinking in clinical risk prediction modeling," *BMC Medical Research Methodology*, vol. 20, no. 1, p. 179, 2020.

[53] D. Acemoglu, U. Akcigit, and W. R. Kerr, "Innovation network," *Proceedings of the National Academy of Sciences*, vol. 113, no. 41, pp. 11483–11488, 2016.

[54] A. Singh, G. Triulzi, and C. L. Magee, "Technological improvement rate predictions for all technologies: Use of patent data and an extended domain description," *Research Policy*, vol. 50, no. 9, p. 104294, 2021.

[55] C. L. Benson and C. L. Magee, "Technology structural implications from the extension of a patent search method," *Scientometrics*, vol. 102, no. 3, pp. 1965–1985, 2015.

[56] C. L. Benson and C. L. Magee, "A hybrid keyword and patent class methodology for selecting relevant sets of patents for a technological field," *Scientometrics*, vol. 96, no. 1, pp. 69–82, 2013.

[57] C. Singh, E. Barme, R. Ward, L. Tupikina, and M. Santolini, "Quantifying the rise and fall of scientific fields," *arXiv preprint arXiv:2107.03749*, 2021.

[58] W. B. Arthur, "Foundations of complexity economics," *Nature Reviews Physics*, vol. 3, no. 2, pp. 136–145, 2021.

[59] C. A. Hidalgo and R. Hausmann, "The building blocks of economic complexity," *Proceedings of the National Academy of Sciences*, vol. 106, no. 26, pp. 10570–10575, 2009.

[60] S. Redner, "Citation statistics from more than a century of physical review," *arXiv*, July 2004.

[61] S. Mukherjee, D. M. Romero, B. Jones, and B. Uzzi, "The nearly universal link between the age of past knowledge and tomorrow's breakthroughs in science and technology: The hotspot," *Science Advances*, vol. 3, apr 2017.

[62] M. Golosovsky and S. Solomon, "Growing complex network of citations of scientific papers: Modeling and measurements," *Physical Review E*, vol. 95, p. 012324, jan 2017.

[63] M. Golosovsky, *Citation Analysis and Dynamics of Citation Networks.* Springer International Publishing, Oct. 2019.

[64] M. Simkin and V. Roychowdhury, "Read before you cite!," *Complex Systems*, vol. 14, pp. 269–274, 2003.

[65] M. V. Simkin and V. P. Roychowdhury, "Stochastic modeling of citation slips," *Scientometrics*, vol. 62, pp. 367–384, Jan. 2005.

[66] S. Goldberg, H. Anthony, and T. Evans, "Modelling citation networks," *Scientometrics*, vol. 105, pp. 1577–1604, December 2015.

[67] N. P. Hummon and P. Dereian, "Connectivity in a citation network: The development of dna theory," *Social networks*, vol. 11, no. 1, pp. 39–63, 1989.

[68] N. P. Hummon and K. Carley, "Social networks as normal science," *Social Networks*, vol. 15, pp. 71–106, Mar 1993.

[69] S. Moore, V. Haines, P. Hawe, and A. Shiell, "Lost in translation: a genealogy of the "social capital" concept in public health," *Journal of Epidemiology & Community Health*, vol. 60, no. 8, pp. 729–734, 2006.

[70] A. Mina, R. Ramlogan, G. Tampubolon, and J. S. Metcalfe, "Mapping evolutionary trajectories: Applications to the growth and transformation of medical knowledge," *Research policy*, vol. 36, no. 5, pp. 789–806, 2007.

[71] C. Calero-Medina and E. C. Noyons, "Combining mapping and citation network analysis for a better understanding of the scientific development: The case of the absorptive capacity field," *Journal of Informetrics*, vol. 2, no. 4, pp. 272–279, 2008.

[72] D. Lucio-Arias and L. Leydesdorff, "Main-path analysis and path-dependent transitions in histcite^{TM}-based historiograms," *Journal of the American Society for Information Science and Technology*, vol. 59, no. 12, pp. 1948–1962, 2008.

[73] J. S. Liu and L. Y. Lu, "An integrated approach for main path analysis: Development of the hirsch index as an example," *Journal of the American Society for Information Science and Technology*, vol. 63, no. 3, pp. 528–542, 2012.

[74] J. C. Ho, E.-C. Saw, L. Y. Lu, and J. S. Liu, "Technological barriers and research trends in fuel cell technologies: A citation network analysis," *Technological Forecasting and Social Change*, vol. 82, pp. 66–79, 2014.

[75] D. Maltseva and V. Batagelj, "Social network analysis as a field of invasions: bibliographic approach to study sna development," *Scientometrics*, vol. 121, no. 2, pp. 1085–1128, 2019.

[76] S.-C. Hung, J. S. Liu, L. Y. Lu, and Y.-C. Tseng, "Technological change in lithium iron phosphate battery: the key-route main path analysis," *Scientometrics*, vol. 100, no. 1, pp. 97–120, 2014.

[77] E. H. Kim, Y. K. Jeong, Y. Kim, and M. Song, "Exploring scientific trajectories of a large-scale dataset using topic-integrated path extraction," *Journal of Informetrics*, vol. 16, no. 1, p. 101242, 2022.

[78] E. Alessandri, "Identifying technological trajectories in the mining sector using patent citation networks," *Resources Policy*, vol. 80, p. 103130, jan 2023.

[79] B. Verspagen, "Mapping technological trajectories as patent citation networks: A study on the history of fuel cell research," *Advances in complex systems*, vol. 10, no. 01, pp. 93–115, 2007.

[80] R. Fontana, A. Nuvolari, and B. Verspagen, "Mapping technological trajectories as patent citation networks. an application to data communication standards," *Economics of innovation and new technology*, vol. 18, no. 4, pp. 311–336, 2009.

[81] D. Barberá-Tomás, F. Jiménez-Sáez, and I. Castelló-Molina, "Mapping the importance of the real world: The validity of connectivity analysis of patent citations networks," *Research policy*, vol. 40, no. 3, pp. 473–486, 2011.

[82] A. Martinelli, "An emerging paradigm or just another trajectory? understanding the nature of technological changes using engineering heuristics in the telecommunications switching industry," *Research policy*, vol. 41, no. 2, pp. 414–429, 2012.

[83] M. Epicoco, "Knowledge patterns and sources of leadership: Mapping the semiconductor miniaturization trajectory," *Research Policy*, vol. 42, no. 1, pp. 180–195, 2013.

[84] Y. Lin, J. Chen, and Y. Chen, "Backbone of technology evolution in the modern era automobile industry: An analysis by the patents citation network," *Journal of Systems Science and Systems Engineering*, vol. 20, no. 4, pp. 416–442, 2011.

[85] J. Kim and J. Shin, "Mapping extended technological trajectories: integration of main path, derivative paths, and technology junctures," *Scientometrics*, vol. 116, no. 3, pp. 1439–1459, 2018.

[86] F. Filippin, "Do main paths reflect technological trajectories? applying main path analysis to the semiconductor manufacturing industry," *Scientometrics*, vol. 126, no. 8, pp. 6443–6477, 2021.

[87] J. R. Clough and T. S. Evans, "What is the dimension of citation space?," *Physica A*, vol. 448, pp. 235–247, 2016.

[88] J. Alcácer, M. Gittelman, and B. Sampat, "Applicant and examiner citations in u.s. patents: An overview and analysis," *Research Policy*, vol. 38, no. 2, pp. 415–427, 2009.
